# The effect of dosing strategies on the therapeutic efficacy of artesunate-amodiaquine for uncomplicated malaria: a meta-analysis of individual patient data

**DOI:** 10.1186/s12916-015-0301-z

**Published:** 2015-03-31

**Authors:** The WorldWide Antimalarial Resistance Network (WWARN) AS-AQ Study Group

**Affiliations:** Worldwide Antimalarial Resistance Network, Centre for Tropical Medicine, Nuffield Department of Clinical Medicine, University of Oxford, Oxford, UK

**Keywords:** Malaria, Plasmodium falciparum, Drug resistance, Artesunate, Amodiaquine, Dosing, Efficacy

## Abstract

**Background:**

Artesunate-amodiaquine (AS-AQ) is one of the most widely used artemisinin-based combination therapies (ACTs) to treat uncomplicated *Plasmodium falciparum* malaria in Africa. We investigated the impact of different dosing strategies on the efficacy of this combination for the treatment of falciparum malaria.

**Methods:**

Individual patient data from AS-AQ clinical trials were pooled using the WorldWide Antimalarial Resistance Network (WWARN) standardised methodology. Risk factors for treatment failure were identified using a Cox regression model with shared frailty across study sites.

**Results:**

Forty-three studies representing 9,106 treatments from 1999-2012 were included in the analysis; 4,138 (45.4%) treatments were with a fixed dose combination with an AQ target dose of 30 mg/kg (FDC), 1,293 (14.2%) with a non-fixed dose combination with an AQ target dose of 25 mg/kg (loose NFDC-25), 2,418 (26.6%) with a non-fixed dose combination with an AQ target dose of 30 mg/kg (loose NFDC-30), and the remaining 1,257 (13.8%) with a co-blistered non-fixed dose combination with an AQ target dose of 30 mg/kg (co-blistered NFDC). The median dose of AQ administered was 32.1 mg/kg [IQR: 25.9-38.2], the highest dose being administered to patients treated with co-blistered NFDC (median = 35.3 mg/kg [IQR: 30.6-43.7]) and the lowest to those treated with loose NFDC-25 (median = 25.0 mg/kg [IQR: 22.7-25.0]). Patients treated with FDC received a median dose of 32.4 mg/kg [IQR: 27-39.0]. After adjusting for reinfections, the corrected antimalarial efficacy on day 28 after treatment was similar for co-blistered NFDC (97.9% [95% confidence interval (CI): 97.0-98.8%]) and FDC (98.1% [95% CI: 97.6%-98.5%]; *P* = 0.799), but significantly lower for the loose NFDC-25 (93.4% [95% CI: 91.9%-94.9%]), and loose NFDC-30 (95.0% [95% CI: 94.1%-95.9%]) (*P* < 0.001 for all comparisons). After controlling for age, AQ dose, baseline parasitemia and region; treatment with loose NFDC-25 was associated with a 3.5-fold greater risk of recrudescence by day 28 (adjusted hazard ratio, AHR = 3.51 [95% CI: 2.02-6.12], *P* < 0.001) compared to FDC, and treatment with loose NFDC-30 was associated with a higher risk of recrudescence at only three sites.

**Conclusions:**

There was substantial variation in the total dose of amodiaquine administered in different AS-AQ combination regimens. Fixed dose AS-AQ combinations ensure optimal dosing and provide higher antimalarial treatment efficacy than the loose individual tablets in all age categories.

**Electronic supplementary material:**

The online version of this article (doi:10.1186/s12916-015-0301-z) contains supplementary material, which is available to authorized users.

## Background

The prompt and effective treatment of confirmed cases of malaria is a key component of all malaria control and elimination programmes [1]. Artemisinin-based combination therapies (ACTs) have become the treatment of choice for uncomplicated *P. falciparum* malaria, and during the last decade have been adopted as first line treatment in most malaria endemic countries [2]. ACTs achieve rapid parasite clearance and have been shown to have high cure rates, and because of the different modes of action of ACT components, the combinations should slow the emergence and spread of drug resistance [3].

Artesunate-amodiaquine (AS-AQ) is currently the first line treatment in 24 countries, mainly in sub-Saharan Africa, and the second most widely used ACT globally after artemether-lumefantrine [2]. AS-AQ is available in three formulations: non-fixed dose combinations (NFDC) either as loose NFDC or as co-blistered NFDC, and as a fixed dose combination (FDC). The efficacy of AS-AQ has been evaluated in a range of epidemiological settings, and although high cure rates have been reported in several studies [4,5], some studies have reported low efficacy rates [6-11]. It has been suggested that the reduced efficacy observed with AS-AQ in some trials is due to amodiaquine resistance selected by prior use of AQ monotherapy, mainly in East Africa [12-14] and Asia [6,7,13,14]. However, the efficacy of AS-AQ has varied between clinical trials even within the same regions [5,15,16], suggesting that different designs and methodology of clinical trials or other confounding factors are responsible for the varying treatment efficacy.

There is variability in dosing regimens between the different formulations of AS-AQ currently available on the market [17]. In particular, young children are vulnerable to suboptimal dosing, since treatment with both co-blistered and loose NFDC in these patients often requires administration of fractions of whole tablets, an issue which is circumvented by the use of pediatric tablets in the fixed dose formulation [18].

In the current analysis, we investigate the spectrum of AS and AQ bodyweight-adjusted (mg/kg) doses administered with the different formulations and assess whether differences in doses or formulations impacted the antimalarial efficacy of AS-AQ.

## Methods

### Data pooling

A systematic review was conducted in PubMed to identify all clinical trials carried out since 1960 with at least one AS-AQ arm in March 2014. All published antimalarial clinical trials published since 1960 were identified by the application of the key terms ((malaria OR plasmod*) AND (amodiaquine OR atovaquone OR artemisinin OR arteether OR artesunate OR artemether OR artemotil OR azithromycin OR artekin OR chloroquine OR chlorproguanil OR cycloguanil OR clindamycin OR coartem OR dapsone OR dihydroartemisinin OR duo-cotecxin OR doxycycline OR halofantrine OR lumefantrine OR lariam OR malarone OR mefloquine OR naphthoquine OR naphthoquinone OR piperaquine OR primaquine OR proguanil OR pyrimethamine OR pyronaridine OR quinidine OR quinine OR riamet OR sulphadoxine OR tetracycline OR tafenoquine)) through the PubMed library. All references containing any mention of antimalarial drugs were tabulated and manually checked to confirm prospective clinical trials. Studies on prevention or prophylaxis, reviews, animal studies or studies of patients with severe malaria were excluded. Further details of the publications or protocols when available were reviewed, and basic details on the study methodology, treatment arms assessed and the study locations documented. These are provided in the WorldWide Antimalarial Resistance Network (WWARN) publication library [19]. Specific details of the studies with at least one AS-AQ arm are available in Additional file 1: Text S1 and Additional file 2: Text S2.

The year of the study was taken as the year in which the paper was published, although the start and end dates of patient enrolment were also recorded. All research groups in the systematic review were contacted to share their data with WWARN, and those who have contributed to the WWARN data repository were also asked whether they were aware of any unpublished or ongoing clinical trials involving AS-AQ, and also asked to contribute those unpublished data if available. Individual study protocol details were available for all trials, either from the publication or as a metafile submitted with the raw data. The WWARN invited investigators to participate in this meta-analysis if their studies included: i) prospective clinical efficacy studies of the treatment of *Plasmodium falciparum* (either alone or mixed infections), ii) treatment with AS-AQ with a minimum of 28 days of follow-up, iii) data available on exact dosages of AS and AQ and iv) PCR genotyping results to determine whether recurrences were due to recrudescence or new infection. Individual patient data from eligible studies were shared; collated and standardised using previously described methodology [20].

### Ethical approval

All data included in this analysis were obtained after ethical approvals from the countries of origin. Ethical approval to conduct individual participant data meta-analyses was granted by the Oxford Tropical Research Ethics Committee (OxTREC), and OxTREC ruled that appropriate informed consent has been met by each study.

### Dosing calculation

The doses of AS and AQ received were calculated from the number of daily tablets administered to each patient. Doses were back-calculated where tablet counts were not available, using the dosing scheme available from study protocols. Only patients completing a full three-day treatment regimen according to the principal investigator and included in the original analysis were included in the meta-analysis. The method of dose calculation was tested as a covariate for risks associated with primary and secondary endpoints, and its influence in the remaining model parameters was explored when found significant.

### Classification of study sites in transmission zones

The study sites were classified into three categories: low, moderate and high malaria transmission intensity based on transmission estimates from the Malaria Atlas Project [21]. More information about this classification is available in Additional file 3: Text S3.

### Statistical analysis

All statistical analyses were carried out based on an *a priori* statistical plan [22], available in Additional file 4: Text S4. The primary endpoint used in this analysis was the PCR-adjusted risk of *P. falciparum* recrudescence at day 28. Secondary endpoints included PCR-adjusted risk of *P. falciparum* recrudescence at day 42, PCR-adjusted risk of new *P. falciparum* infection, and parasite positivity rates (PPRs) on days 1, 2 and 3 after treatment initiation. The overall efficacy at day 28 and day 42 was computed using survival analysis [Kaplan-Meier (K-M) estimates]; comparisons of K-M survival curves were performed using log rank tests stratified by study site (using a combination of trial and study site). Gehan’s test was used when K-M curves crossed. Definitions of outcome and censoring are detailed in the WWARN Clinical Module DMSAP v1.2 available in Additional file 5: Text S5 [23]. The mg/kg dose of AQ was considered as the primary risk factor for recrudescence because of the longer half-life of its active metabolite desethylamodiaquine. The dose of AS was considered as the primary risk factor for early parasitological response due to its more rapid anti-parasitic activity and its shorter half-life. Risk factors for PCR-confirmed recrudescence and new infections were analysed using a Cox proportional hazards regression with shared frailty across study sites to account for any unobserved heterogeneity [24,25]. Known confounders (age, baseline parasitemia, region and mg/kg dose) were kept in the model regardless of statistical significance. Any other variables significant at the 10% level in the univariable analysis were retained for multivariable analysis; the inclusion of each significant variable in the final model was based on a likelihood ratio test assessed at the 5% level of significance. Cox-Snell’s and martingale residuals were examined to assess the model fit; the underlying assumption of proportional hazards was tested and reported when violated. The population attributable risks (PARs) associated with the risk factors in the final model were calculated based on their prevalence in the study data and adjusted hazard ratio (AHR) using [prevalence × (AHR-1)]/ {1 + [prevalence × (AHR-1)]} [26]. The overall PAR (for a combination of factors), which is non-additive, was calculated as 1-[(1-PAR_1_) × (1-PAR_2_) × … × (1-PAR_n_)].

Risk factors associated with PPRs were assessed using logistic regression with study sites fitted as a random effect. The relationship between drug dose and gastrointestinal side effects (vomiting and diarrhea), anemia and neutropenia was also explored using mixed effects logistic regression with random effects specified for study sites. Proportions were compared using chi-squared tests or Fisher’s exact tests when samples were small. Non-normal data were compared with the Mann-Whitney U test. The assessment of bias where individual patient data were not available for analysis was performed using a simulation approach, based on the data included in the analysis. PCR-corrected efficacy estimates (*θ*) at day 28 for the given age range for the studies not available were estimated from the available data. A total of *n* (*n* = study sample size) patients were simulated from a binomial distribution (assuming a simple case of no censoring structure) with probability of success, *θ*_*i*_. A study with a sample size *n* was then simulated 1,000 times from which the mean cure rate and associated 95% CI were estimated. When the observed cure rate for the non-available study fell within the simulated 95% CI, it was concluded that excluded studies were similar to the studies in the meta-analysis. All statistical analyses were carried out in R (Version 2.14.0, The R Foundation for Statistical Computing) using *survival* and *lme4* packages.

## Results

### Characteristics of included studies

**Figure 1:**
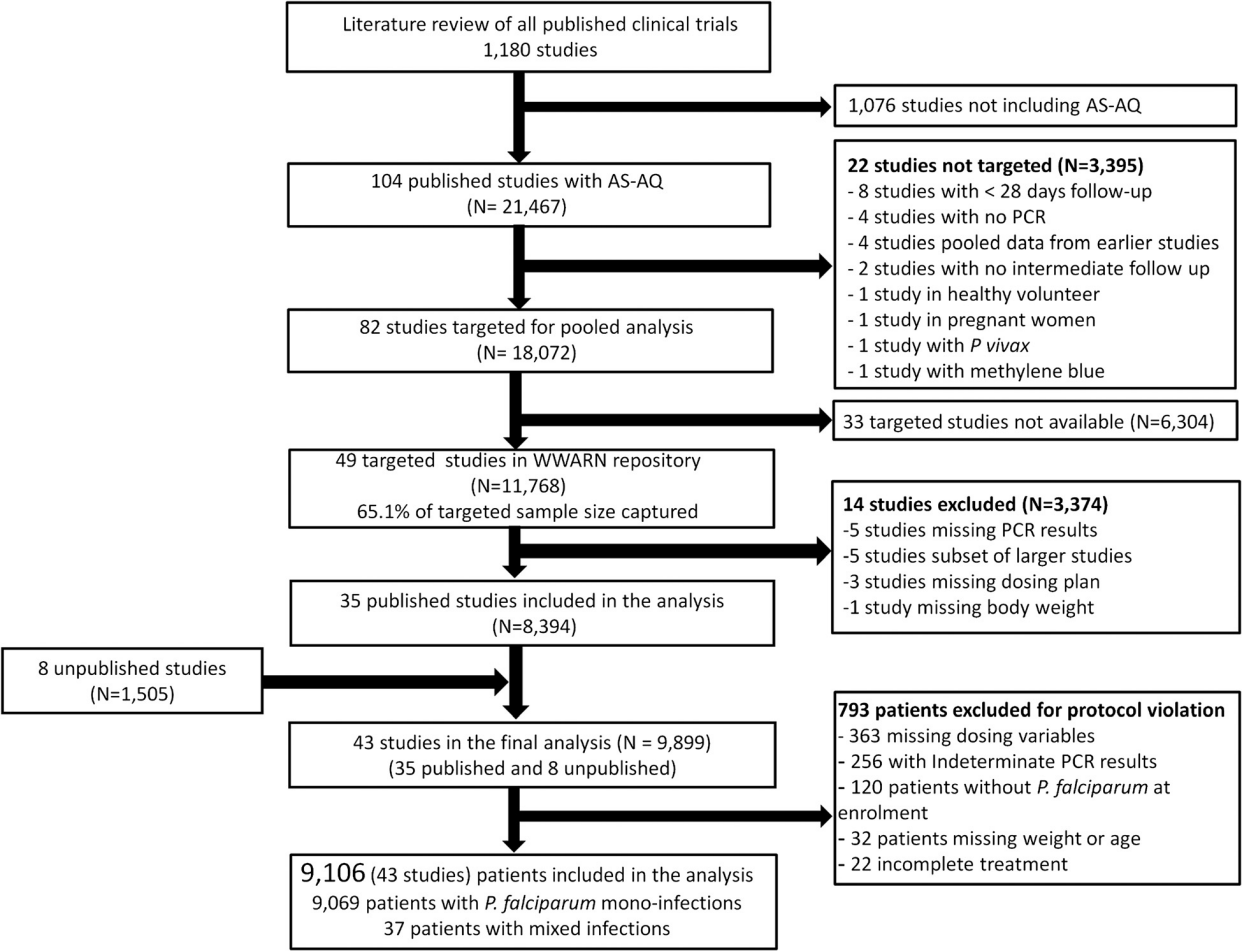
Patient flowchart.

Data were available from 57 studies (13,273 treatments), including 8 unpublished studies (1,505 treatments) and 49 published studies (11,768 treatments), representing 65.1% of the targeted published literature (18,072 treatments). Fourteen studies (3,374 treatments) did not meet the inclusion criteria and an additional 793 treatments were excluded for a variety of protocol violations, of which 2.8% (22/793) did not include the full course of treatment (Figure 1). In total, 43 studies (9,106 treatments) were included in the final analysis, of which 39 (8,635 treatments) were conducted in Africa between 1999 and 2012, 1 in South America in 2000 (37 treatments) and the remaining 3 studies (434 treatments) in Asia between 2005 and 2009 (Table 1). Overall, 13 studies (2,106 treatments) were conducted in areas of high malaria transmission intensity, 13 (2,958 treatments) in areas of moderate transmission, and 11 (1,219 treatments) in areas of low transmission, and the remaining 6 studies included sites with varied transmission intensity (2,823 treatments). Patients were followed for 28 days in 34 studies (7,865 treatments), for 35 days in 1 study (82 treatments), for 42 days in 7 studies (1,017 treatments) and for 63 days in 1 study (142 treatments). Parasite genotyping of recurrent infections was carried out in all studies; with 5 studies (1,257 treatments) using a single marker (MSP2 or MSP1); 16 studies (2,862 treatments) using two markers (MSP1 and MSP2); 16 studies (3,768 treatments) using three markers (MSP1, MSP2 and GLURP); 3 studies (898 treatments) using MSP1, MSP2 and microsatellites; 1 study using microsatellites only (13 treatments); the genotyping method was not stated in 1 study (276 treatments) and genotyping was not carried out in 1 study with no recurrences (32 treatments).

**Table 1:** **Studies included in the meta-analysis**

**Study** ^**a**^	**Number of patients treated with AS-AQ**	**Country**	**Age range (months)**	**Target dose (mg/kg) for artesunate & amodiaquine**	**Manufacturer**	**Formulation**	**Supervision**	**Reference**
**Adjuik-2002**	390	Multicentric	6-59	12 & 30	Sanofi-Synthélabo & Parke-Davis	Loose NFDC	Full	[8]
**Anvikar-2012** ^**b**^	199	India	6-720	12 & 30	Sanofi-Aventis	FDC	Full	[36]
**Barennes-2004**	32	Burkina Faso	12-180	12 & 30	Sanofi Winthrop AMO & Hoechst Marion Roussel	Loose NFDC	Full	[64]
**Bonnet-2007**	110	Guinea	6-59	12 & 30	Guilin Pharmaceutical & Parke-Davis	Loose NFDC	Full	[65]
**Brasseur-2009** ^**b**^	276	Senegal	All ages	N/A	Sanofi-Aventis	Co-blistered NFDC	Full/partial^e^	[17]
**Bukirwa-2006**	203	Uganda	12-120	12 & 25	Sanofi-Aventis & Parke-Davis, Pfizer	Loose NFDC	Full	[66]
**Dorsey-2007**	145	Uganda	12-120	12 & 25	Sanofi-Aventis & Pfizer	Loose NFDC	Full	[67]
**Espié-2012**	149	DRC	6-59	12 & 30	Sanofi-Aventis	FDC	Full	[34]
**Faucher-2009**	94	Benin	6-60	12 & 30	Sanofi-Aventis	FDC	Partial	[68]
**Faye-2010**	155	Multicentric	>84	N/A	Pfizer	Co-blistered NFDC	Full	[69]
**Gaye-2010b** ^**d**^	129	Senegal	12-720	12 & 30	Sanofi-Aventis	FDC	Full	[Unpublished]
**Grandesso-2003** ^**d**^	86	Uganda	6-59	12 & 30	Sanofi & Park-Davis	Loose NFDC	Full	[Unpublished]
**Grandesso-2006**	123	Sierra Leone	6-59	12 & 30	Sanofi Winthrop AMO & Pfizer	Loose NFDC	Full	[44]
**Guthmann-2005**	96	Angola	6-59	12 & 30	Sanofi Winthrop & Parke Davis	Loose NFDC	Full	[70]
**Guthmann-2006**	68	Angola	6-59	12 & 30	Sanofi Winthrop & Parke Davis	Loose NFDC	Full	[71]
**Hamour-2005**	71	Sudan	6-59	12 & 30	Sanofi & Park-Davis	Loose NFDC	Full	[72]
**Hasugian-2007**	93	Indonesia	>12	12 & 30	Guilin Pharmaceuticals & Aventis	Loose NFDC	Full	[6]
**Jullien-2010**	27	Kenya	216-720	N/A	Sanofi-Aventis	Co-blistered NFDC	Full	[73]
**Jullien-2010**	24	Kenya	216-720	12 & 30	Sanofi-Aventis	FDC	Full	[73]
**Juma-2005** ^**d**^	201	Kenya	6-59	12 & 30	Sanofi-Aventis	Loose NFDC	Full	[Unpublished]
**Karema-2006**	251	Rwanda	12-59	12 & 30	Sanofi-Aventis	Loose NFDC	Full	[74]
**Kayentao-2009**	128	Mali	6-59	12 & 30	-	Co-blistered NFDC	Full	[75]
**Laminou-2011** ^**d**^	80	Niger	6-180	12 & 30	Sanofi-Aventis	FDC	Partial	[Unpublished]
**Mårtensson-2005**	202	Tanzania	6-59	12 & 30	Mepha & Roussel	Loose NFDC	Full	[45]
**Menan-2012** ^**d**^	110	Ivory Coast	12-480	12 & 30	Sanofi-Aventis	FDC	Full	[Unpublished]
**Menard-2008**	332	Madagascar	6-180	12 & 30	-	Loose NFDC	Full	[76]
**Ndiaye-2009**	625	Multicentric	All ages	12 & 30	Sanofi-Aventis	FDC	Full	[30]
**Ndiaye-2011**	179	Senegal	All ages	12 & 30	Sanofi-Aventis	FDC	Full^f^	[32]
**Nikiema-2010** ^**d**^	527	Burkina Faso	6-120	12 & 30	Sanofi-Aventis	FDC	Full	[Unpublished]
**Osorio-2007**	37	Columbia	12-780	12 & 30	Sanofi-Aventis	Loose NFDC	Full	[77]
**Rwagacondo-2004**	157	Rwanda	6-59	12 & 30	Dafra	Loose NFDC	Full	[11]
**Sagara-2012**	230	Mali	≥6	12 & 30	Sanofi-Aventis	Co-blistered NFDC	Full	[78]
**Sanofi-2013** ^**d**^	203	Uganda	6-59	12 & 30	Sanofi-Aventis	FDC	Full^f^	[Unpublished]
**Schramm-2013**	147	Liberia	6-72	12 & 30	Sanofi-Aventis	FDC	Full	[38]
**Sinou-2009** ^**c**^	13	Congo	≥192	12 & 30	Saokim Pharmaceuticals Co	FDC	Full	[31]
**Sirima-2009** ^**b**^	441	Burkina Faso	6-59	12 & 30	Sanofi-Aventis	Co-blistered NFDC	Full	[18]
**Sirima-2009** ^**b**^	437	Burkina Faso	6-59	12 & 30	Sanofi-Aventis	FDC	Full	[18]
**Smithuis-2010**	142	Myanmar	>6	12 & 32/4	Sanofi-Aventis	FDC	Partial	[7]
**Staedke-2004**	130	Uganda	6-120	12 & 25	Sanofi-Pfizer	Loose NFDC	Full	[79]
**Swarthout-2006**	82	DRC	6-59	12 & 30	Sanofi and Parke Davis & Pfizer	Loose NFDC	Full	[80]
**Temu-2010** ^**d**^	99	Liberia	6-60	12 & 30	Sanofi-Aventis	FDC	Full	[Unpublished]
**The 4ABC StudyGroup-2011**	981	Multicentric	6-59	12 & 30	Sanofi-Aventis	FDC	Full	[15]
**Thwing-2009**	101	Kenya	6-59	12 & 25	Cosmo Pharmaceuticals & Pfizer	Loose NFDC	Full	[46]
**van den Broek-2006**	87	Congo	6-59	12 & 30	Cosmo Pharmaceuticals & Pfizer	Loose NFDC	Full	[81]
**Yeka-2005**	714	Uganda	≥6	12 & 25	Sanofi-Pfizer	Loose NFDC	Full	[82]

^a^Full details of the references and study design are available in Additional file 1: Text S1.

^b^The dose was given based on age bands for these studies. For the rest of the studies, dosing was based on weight categories.

^c^All patients recruited given 2 doses/day.

^d^These studies are unpublished.

^e^Fully supervised between 2002-2004 and partially supervised in 2005.

^f^The first episodes of malaria were fully supervised in these studies.

### Drug formulations

Three different formulations from nine different manufacturers were used in the 43 studies included in this analysis (Table 1). Overall, 15 studies (3,677 treatments) used FDC, 22 (3,711 treatments) used loose NFDC, 4 studies (789 treatments) used co-blistered NFDC and 2 studies (929 treatments) compared co-blistered NFDC to FDC (Table 1). Various tablet strengths were included in the different formulations (Table 2). However, only FDC had pediatric tablets (Table 2 and Additional file 1: Text S1). All the studies using FDC and co-blistered NFDC and some studies using loose NFDC with a target dose of 30 mg/kg amodiaquine (loose NFDC-30) administered identical doses of AS and AQ on each of the three days of treatment, with a target dose of 4 mg/kg/day for AS and 10 mg/kg/day for AQ (Additional file 1: Text S1). However, other studies administering a loose NFDC with a target dose of 25 mg/kg AQ (loose NFDC-25) gave a higher daily AQ dose on day 1 and 2 (10 mg/kg/day) and a lower AQ dose on day 3 (5 mg/kg/day), while the AS dose (4 mg/kg/day) was similar over the three days (Additional file 1: Text S1).

**Table 2:** **Tablet strengths of the different formulations**

**Formulation**	**Tablet strength**			
	**Pediatric formulation**		**Adult formulation**	
	**AQ**	**AS**	**AQ**	**AS**
**Loose NFDC**	-	-	200 mg	50 mg
**Co-blistered NFDC**	-	-	153 mg	50 mg
**FDC (Trimalact®)**	-	-	300 mg	100 mg
**FDC (Coarsucam®/Winthrop®)**	67.5 mg	25 mg	270 mg	100 mg
	135 mg	50 mg		

### Baseline characteristics

The patient baseline characteristics are summarised in Table 3. Overall 8.6% (783/9,106) of patients were less than one year of age, 62.1% (5,653/9,106) were from 1 to 5 years of age, 16.9% (1,535/9,106) from 5 to 12 years and 12.5% (1,135/9,106) 12 years or older. The overall median age was 3.0 years [IQR: 1.8-6.0, range: 0.0-80.0], with patients from Africa being significantly younger (median 3.0 years, [IQR: 1.7-5.0, range: 0.0-80.0]) than those from Asia (median 17.0 years, [IQR: 8.0-28.0, range: 0.6-80.0] or South America (median 20.0 years, [IQR: 16-25, range: 8.0-58.0]) (Table 2). At enrolment, 56.6% (3,908/6,906) of the patients were anemic (Hb < 10 g/dl) and 11% (527/4,796) had patent gametocytemia based on blood smears, with significant regional differences (Table 3).

**Table 3:** **Patient characteristics at baseline**

**Variable**	**Asia**	**Africa**	**South America** ^**a**^	**Overall**
N	434 (4.77%)	8635 (94.83%)	37 (0.41%)	9106
Study period	2005-2009	1999-2012	2000-2004	1999-2012
**Gender**				
Female	38.7% [168/434]	47.0% [4,060/8,635]	18.9% [7/37]	46.5% [4,235/9,106]
**Age**				
Median age [IQR, range] in years	17 [8-28,0.6-80]	3 [1.7-5,0-80]	20 [16-25,8-58]	3 [1.8-6, 0-80]
<1 y	0.2% [1/434]	9.1% [782/8,635]	0.0% [0/37]	8.6% [783/9,106]
1 to <5 y	7.8% [34/434]	65.1% [5,619/8,635]	0% [0/37]	62.1% [5,653/9,106]
5 to <12 y	25.3% [110/434]	16.5% [1,421/8,635]	10.8% [4/37]	16.9% [1,535/9,106]
≥12 y	66.6% [289/434]	9.4% [813/8,635]	89.2% [33/37]	12.5% [1,135/9,106]
**Treatment supervision** ^**b**^				
Full	67.3% [292/434]	95.1% [8,212/8,635]	100.0% [37/37]	93.8% [8,541/9,106]
Partial	32.7% [142/434]	4.9% [423/8,635]	0.0% [0/37]	6.2% [565/9,106]
**Drug formulation**				
Fixed dose combination (FDC)	78.6% [341/434]	44.0% [3,797/8,635]	0.0% [0/37]	45.4% [4,138/9,106]
Co-blistered non-fixed dose combination (co-blistered NFDC)	0.0% [0/434]	14.6% [1,257/8,635]	0.0% [0/37]	13.8% [1,257/9,106]
Loose non-fixed dose combination: target dose 25 mg/kg (loose NFDC-25)	0.0% [0/434]	15.0% [1,293/8,635]	0.0% [0/37]	14.2% [1,293/9,106]
Loose non-fixed dose combination: target dose 30 mg/kg (loose NFDC-30)	21.4% [93/434]	26.5% [2,288/8,635]	100.0% [37/37]	26.6% [2,418/9,106]
**Enrolment clinical variables**				
Geometric mean parasitemia [95% CI] in parasites/μl	8,504 [7,409-9,761]	19,508 [18,944-20,089]	80 [55-116]	18,338 [17,801-18,891]
Median weight [IQR, range] in kg	40 [20-50,7-72]	12 [10-17, 5-104]	59 [47-65,24-80]	12.7 [10-18, 5-104]
Underweight for age^c^	37.1% [13/35]	20.6% [1,248/5,821]	-	20.7% [1,297/6,269]
Anemic (hb < 10 g/dl)^d^	34.3% [149/434]	59% [3,754/5,821]	13.5% [5/37]	56.6% [3,908/6,906]
Gametocytes presence^e^	39.4% [56/142]	10.0% [462/5,821]	24.3% [9/37]	11.0% [527/4,796]
Fever (temp > 37.5 °C)	77.7% [227/292]	66.4% [5,769/5,821]	16.2% [6/37]	68.5% [6,002/8,766]
Hemoglobin [mean ± SD] in g/dl	10.9 ± 2.29	9.5 ± 2.06	12.06 ± 1.93	9.6 ± 2.11

^a^Single study from Columbia.

^b^Treatment supervision: The treatment was fully supervised if each dose of the three-day regimen was administered by a nurse/or any other medical staff. The treatment was partially supervised if only the dose on the first day was administered by medical staff, the dose on day 2 and day 3 being self-administered by the patients or the parents/guardians.

^c^Defined using a weight-for-age score (WAZ) < -2 in children <5 years of age. WAZ scores outside the range (-6.6) were treated as outliers.

^d^Asia v Africa (*P* = 0.005), Asia v South America (*P* = 0.438) and Africa v South America (*P* = 0.042).

^e^Asia v Africa (*P* < 0.001), Asia v South America (*P* = 0.236) and Africa v South America (*P* = 0.308).

### Distribution of AQ and AS dosing

**Figure 2:**
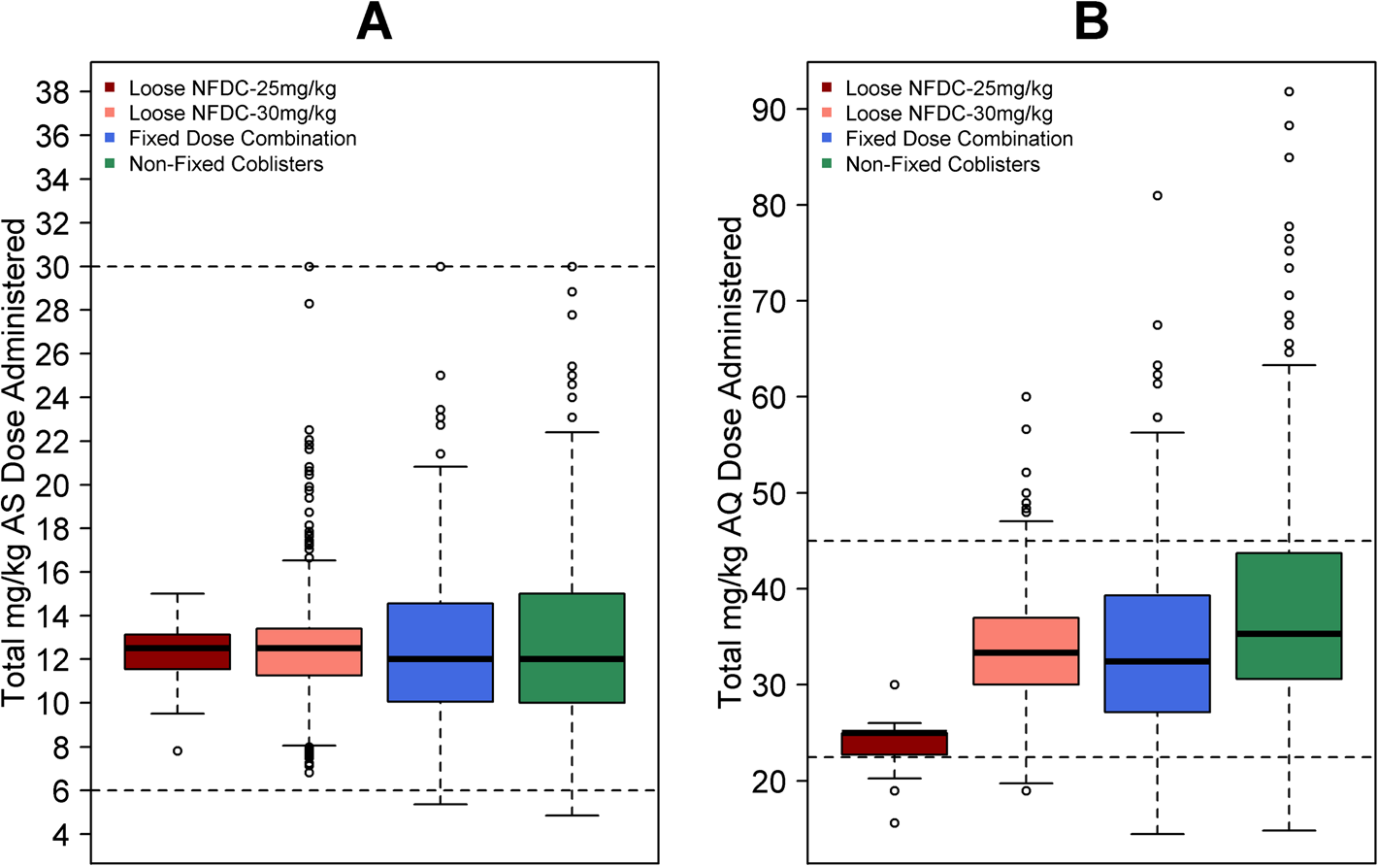
Total mg/kg dose for artesunate **(A)** and amodiaquine **(B)**. The dotted line represents the WHO therapeutic dose range for artesunate (6 to 30 mg/kg) and amodiaquine (22.5 to 45 mg/kg).

Overall, the median dose of AQ was 32.1 mg/kg [IQR: 25.9-38.2], with the highest AQ doses administered to patients treated with co-blistered NFDC and the lowest to those administered loose NFDC-25. The latter group received a median dose of 25 mg/kg [IQR: 22.7-25.0], which was significantly lower than the dose received in the FDC (median = 32.4 mg/kg [IQR: 27.0-39.0]) (*P* < 0.001) and co-blistered NFDC (median = 35.3 mg/kg [IQR: 30.6-43.7]) (*P* < 0.001) groups. Patients treated with loose NFDC-30 received a median dose of 33.7 mg/kg [IQR: 30.6-38.1], similar to that received by patients treated with FDC, but significantly lower compared to patients treated with co-blistered NFDC (*P* < 0.001). Patients younger than 1 year received a lower dose of AQ (median = 28.9 mg/kg [IQR: 25.0-35.1]) compared to the other age categories (*P < 0.001* for all comparisons), except for the patients treated with loose NFDC-30, for whom the dose was similar across the different age groups (*P* = 0.91) (Table 3). All patients (3,711 treatments) treated with loose NFDCs were dosed based on body weight; 85% (3,502/4,138) of patients receiving FDC were dosed based on body weight and 15% (636/4,138) based on age; and 69% (872/1,257) of patients treated with co-blistered NFDC were dosed based on body weight and 31% (385/1,257) based on age. Overall, only 3.4% (309/9,106) of patients received a total AQ dose below 22.5 mg/kg, the lower bound of the currently recommended WHO therapeutic range (22.5 to 45 mg/kg over three days) [27], most of whom (68%, 211/309) were treated with loose NFDC-25. The proportion of patients receiving an AQ dose below this threshold was 16.3% (211/1,293) in those treated with loose NFDC-25, 1.7% (41/2,418) in those treated with loose NFDC-30, 1.1% (45/4,138) in those treated with FDC and 0.9% (12/1,257) in those treated with co-blistered NFDC. The overall median dose of AS administered was 12.5 mg/kg [IQR: 10.7-13.6], which was similar across diverse formulations and age categories (Table 4 and Figure 2).

**Table 4:** **Total mg/kg dose administered (median [IQR, (range)]) for artesunate and amodiaquine**

	**FDC**	**Co-blistered NFDC**	**Loose NFDC-30**	**Loose NFDC-25**
**Artesunate dose (mg/kg)** ^**a**^				
<1 y	10.7 [9.4-12.5 , 7.5-16.7]	10.7 [9.6-12.3 , 7.5-20.5]	12.5 [10.6-13.7 , 8.3-17.9]	12.5 [10.7-14.1 , 9.5-15]
1 to <5 y	12.5 [10.7-15 , 5.4-30]	13.4 [11.2-15.2 , 4.8-30]	12.6 [11.5-13.6 , 6.8-30]	12.5 [11.3-13.4 , 10.4-14.1]
5 to <12 y	12.5 [10-15 , 7-20]	11.5 [9.7-13.7 , 5.5-21.4]	11.5 [10-12.5 , 6.8-15]	12.5 [11.9-13 , 11.5-13.4]
≥12 y	10.9 [9.5-13 , 5.8-21.4]	10.9 [9.4-13 , 6-24]	11.5 [10.9-12.1 , 7.5-14]	11.7 [11.2-12 , 7.8-12.5]
Overall	12 [10-14.5 , 5.4-30]	12.0 [10-15 , 4.8-30]	12.5 [11.1-13.5 , 6.8-30]	12.5 [11.5-13.1 , 7.8-15]
**Amodiaquine dose (mg/kg)** ^**a**^				
<1 y^b^	28.9 [25.3-33.8 , 20.3-45]	32.6 [28.7-36.5 , 22.8-62.9]	33.9 [30.3-37 , 19-50]	22.9 [21.4-25 , 19-30]
1 to <5 y	33.8 [28.9-40.5 , 14.5-81]	38.3 [32.2-45.9 , 14.8-91.8]	33.3 [30-37.5 , 19.7-60]	25.0 [22.7-25 , 21.1-25]
5 to <12 y	33.8 [27-40.5 , 18.9-54]	35.3 [29.5-42 , 16.7-65.6]	34.1 [31.6-39.8 , 27.3-60]	24.1 [23.7-25 , 22.6-26]
≥12 y	29.5 [25.7-35.2 , 15.6-57.9]	33.4 [28.7-39.9 , 18-73.4]	38.9 [33.3-44.2 , 28.1-55.8]	24.0 [23.1-25 , 15.6-26]
Overall	32.4 [27-39 , 14.5-81]	35.3 [30.6-43.7 , 14.8-91.8]	33.7 [30.6-38.1 , 19-60]	25.0 [22.7-25 , 15.6-30]

^a^The overall median mg/kg amodiaquine dose administered was 32.1 mg/kg [IQR = 25.9-38.2, range = 14.5-91.8].

The overall median mg/kg artesunate dose administered was 12.5 mg/kg [IQR = 10.7-13.6, range = 4.8-30].

^b^In children <1 year, the overall median mg/kg amodiaquine dose administered was 28.9 mg/kg [IQR = 25-35.1, range = 18.9-62.7].

### Early parasitological response

Overall, the early parasitological response to treatment was rapid in those studies. The PPR decreased from 64.7% [95% CI: 58.5-71.0%] on day 1 to 7.1% [95% CI: 5.2-9.0%] on day 2 and 1.0% [95% CI: 0.6-1.4%] on day 3 (Table 1 in Additional file 6: Text S6). High baseline parasitemia was the only independent risk factor associated with remaining parasitemic on day 1, day 2 and day 3 (Table 2 in Additional file 6: Text S6). The overall mg/kg dose of AS was not a significant predictor of parasite positivity on any day for any drug formulation, either in the overall population or in young children.

### Late parasitological response

**Figure 3:**
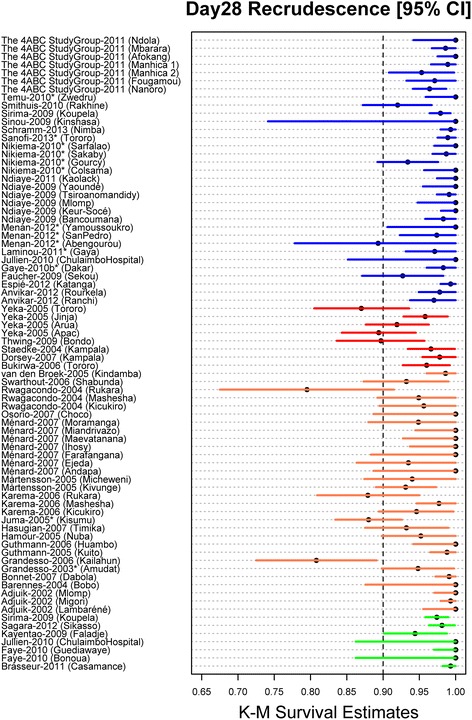
Day 28 survival estimates. PCR adjusted recrudescence estimates on day 28 were generated using Kaplan-Meier method stratified by study sites for loose NFDC-25 [red], loose NFDC-30 [orange], co-blistered NFDC [green] and FDC [blue]. The associated error bars are 95% confidence interval (CI) for survival estimates. 95% CIs were generated using Wilson’s method in case of no failures using the number of patients at risk on day 28. Unpublished studies are represented by *. ** The risk of recrudescence by day 28 was significantly higher in three study sites (Kailahun (Sierra Leone), Kisumu (Kenya) and Rukara (Rwanda)), where patients were treated with loose NFDC-30 compared to the other study sites in the loose NFDC-30 category (hazards ratio (HR) = 6.27 [95% CI:2.40-16.32], *P* < 0.001). Patients treated with loose NFDC-30 in these three sites were at higher risk of recrudescence (HR = 8.40 [95% CI: 3.23-21.83], *P* < 0.001) compared to patients treated with FDC and those treated with co-blistered NFDC (HR = 8.22 [95% CI: 2.66-25.40], *P* < 0.001). The risk of recrudescence was similar between patients treated with loose NFDC-30 in the other sites compared to those treated with FDC (HR = 1.34 [95% CI: 0.77-2.34]; *P* = 0.300) or co-blistered NFDC (HR = 1.31 [95% CI: 0.59-2.87], *P* = 0.500). All the HR was derived from univariable Cox model with study sites fitted as random effect.

In total, 18.2% (1,657/9,106) of the patients had parasitemia detected during follow-up, of whom 295 (3.2%) were PCR-confirmed as recrudescences. Of these PCR-confirmed recrudescences, 276 (93.6%) occurred by day 28 and the remaining 19 (6.4%) between days 28 and 42. The PCR-adjusted clinical efficacy was significantly higher at day 28 in patients treated with FDC (98.1% [95% CI: 97.6-98.5%]) or co-blistered NFDC (97.9% [95% CI: 97-98.8%]) compared to patients treated with either loose NFDC-30 (95.0% [95% CI: 94.1-95.9%]) or loose NFDC-25 (93.4% [95% CI: 91.9-94.9%]); (*P* < 0.001 for all comparisons) (Table 5, Figure 3). At day 28, the efficacy was lowest in infants (<1 year) treated with loose NFDC-25 (90.9% [95% CI: 85.6-96.1%]). In this age category the efficacy of loose NFDC-30 was 93.8% [95% CI: 90.7-96.8] at day 28 and 85.7% [95% CI: 76.6-94.9%] at day 42.

**Table 5:** **PCR-corrected adequate clinical and parasitological response (ACPR) of artesunate-amodiaquine**

	**Survival estimates on day 28** ^**a, b**^								**Survival estimates on day 42** ^**a, b**^			
	**FDC**		**Co-blistered NFDC** ^**c**^		**Loose NFDC-30**		**Loose NFDC-25** ^**c**^		**FDC**		**Loose NFDC-30**	
**Age category**	**At risk**	**K-M [95% CI]**	**At risk**	**K-M [95% CI]**	**At risk**	**K-M [95% CI]**	**At risk**	**K-M [95% CI]**	**At risk**	**K-M [95% CI]**	**At risk**	**K-M [95% CI]**
<1 y	207	97.8 [95.9-99.7]	77	98.7 [96.3-100]	222	93.8 [90.7-96.8]	95	90.9 [85.6-96.1]	42	95.6 [90.9-100]	28	85.7 [76.6-94.9]
1 to <5 y	2,044	97.9 [97.3-98.5]	511	96.9 [95.4-98.3]	1,340	94 [92.8-95.2]	532	92.2 [90.2-94.2]	325	95.7 [94-97.3]	103	92.5 [90-94.9]
5 to <12 y	565	98.1 [97-99.2]	192	98.6 [97-100]	317	98.8 [97.5-100]	211	97.4 [95.3-99.5]	65	95.4 [91.6-99.2]	15	98.8 [97.5-100]
≥12 y	570	98.6 [97.6-99.5]	203	99.6 [98.9-100]	140	98.6 [96.7-100]	31	100 [88.9-100.0]^d^	142	97.9 [96.3-99.5]	34	93.2 [86.2-100]
**Region**												
West Africa	2,167	98.1 [97.6-98.7]	959	97.8 [96.9-98.7]	337	94.9 [92.6-97.2]	-	-	257	95.3 [93.3-97.3]	-	-
East Africa	299	98.9 [97.8-100]	24	100 [100-100]	921	92.8 [91.2-94.4]	869	93.4 [91.9-94.9]	81	98.9 [97.8-100]	127	89.5 [86.3-92.7]
Rest of Africa	615	98.6 [97.8-99.5]	-	-	664	98.3 [97.3-99.2]	-	-	124	97.1 [94.8-99.4]	-	-
Asia	305	95.5 [93.2-97.7]	-	-	69	93.2 [87.5-99]	-	-	112	93.8 [90.7-97]	53	90.2 [83.3-97.1]
S America	-	-	-	-	30	100 [88.7-100]^**d**^	-	-	-	-	-	-
**Overall**	**3,386**	**98.1 [97.6-98.5]**	**983**	**97.9 [97-98.8]**	**2,021**	**95 [94.1-95.9]**	**869**	**93.4 [91.9-94.9]**	**574**	**96.1 [95-97.3]**	**180**	**92.1 [89.8-94.4]**

^**a**^Kaplan-Meier estimates were generated using all the individual data rather than combining estimates from individual trials. *n* is the number of patients at risk (*n*) on day 28.

^b^Pairwise comparisons at day 28 using the Mantel-Haenszel (log-rank ) test.

FDC v co-blistered NFDC (*P* = 0.799).

FDC v loose NFDC-30 (*P* < 0.001).

FDC v loose NFDC-25 (*P* < 0.001).

Co-blistered NFDC v loose NFDC-30 (*P* < 0.001).

Co-blistered NFDC v loose NFDC-25 (*P* < 0.001).

Loose NFDC-30 v loose NFDC-25 (*P* = 0.036).

^c^Patients followed up only up to 28 days.

^d^Exact confidence intervals using Wilson’s method using number of patients at risk on the given day.

### Risk factors for recrudescence

In univariable analysis, five risk factors on admission were associated with PCR-confirmed recrudescence by day 28: being under 5 years compared to ≥12 years of age, high baseline parasitemia, baseline anemia (Hb < 10 g/dl), and being treated with either loose NFDC-25 or loose NFDC-30 (compared to FDC). There was no significant difference in the efficacy between co-blistered NFDC and FDC (*P* = 0.950). In multivariable analysis, high baseline parasitemia (AHR = 1.39 [95% CI: 1.10-1.74]; *P* = 0.005 per 10-fold increase), being <1 year old (AHR = 3.93 [95% CI: 1.76-8.79]; *P* = 0.001 compared to ≥ 12 years), and being 1 to 5 years old (AHR = 4.47 [95% CI: 2.18-9.19]; *P* < 0.001 compared to ≥ 12 years) were significant risk factors for recrudescence. Patients treated with loose NFDC-25 were at 3.5-fold increased risk of recrudescence (AHR = 3.51 [95% CI: 2.02-6.12]; *P* < 0.001) compared to patients treated with FDC. This category accounted for a quarter (PAR = 25.8%) of all recrudescent infections (Table 6). Patients treated with loose NFDC-30 were not at higher risk of recrudescence compared to patients treated with FDC (Table 6). However, a higher risk of recrudescence was observed in patients treated with loose NFDC-30 in three study sites, in Kenya (Kisumu, n = 201), Sierra Leone (Kailahun, n = 123) and Rwanda (Rukara, n = 137) (AHR = 7.75 [95% CI: 4.07-14.76]; *P* < 0.001, compared to FDC) (Figure 3). Patients from Asia were at seven fold increased risk of recrudescence compared to patients from Africa (AHR = 7.39 [95% CI: 3.45-15.86]; *P* < 0.001). The final model accounted for 92.6% of all recrudescences, with patients 1 to 5 years of age accounting for over two-thirds of all failures, PAR = 69% (Table 6).

**Table 6:** **Univariable and multivariable risk factors for PCR-confirmed recrudescent failures at day 28**

		**Univariable analysis**		**Multivariable analysis** ^**b**^		**Population attributable risk** ^**c**^	
				**(N = 9,058)**			
**Variable**	**Total** ***n*** **[** ***n*** **]** ^**a**^	**Crude HR [95% CI]**	***p*** **-Value**	**Adjusted HR [95% CI]**	***P*** **-Value**	**Freq.**	**PAR**
**Age (y)**	9,095 (265)	0.92 [0.89-0.96]	<0.001	-	-	-	-
**Amodiaquine dose (5 mg/kg)**	9,095 (265)	0.94 [0.84-1.04]	0.220	0.94 [0.84-1.05]	0.280	-	-
**Enrolment clinical variables**							
Parasitemia (per 10-fold)	9,095 (265)	1.46 [1.16-1.84]	0.001	1.39 [1.1-1.74]	0.005	10.4%	3.7%
Parasitemia >100,000 parasites/μl	9,095 (265)	1.41 [0.98-2.05]	0.066	-	-	-	-
Fever (temp > 37.5°C)	8,755 (252)	1.05 [0.78-1.41]	0.760	-	-	-	-
Hemoglobin (g/dl)	6,895 (237)	0.93 [0.87-1.00]	0.055	-	-	-	-
Anemia (Hb < 10 g/dl)	6,895 (237)	1.37 [1.04-1.81]	0.028	-	-	-	-
Gametocytes presence	4,790 (99)	1.04 [0.54-1.98]	0.910	-	-	-	-
Underweight (WAZ < −2)^d^	6,260 (616)	0.87 [0.61-1.26]	0.470	-	-	-	-
**Gender**							
Female (reference)	4,231 (126)	1	-	-	-	-	-
Male	4,702 (124)	0.91 [0.71-1.16]	0.450	-	-	-	-
**Age category**							
≥12 y (reference)	1,135 (12)	1	-	-	-	-	-
<1 y	782 (31)	3.15 [1.46-6.78]	0.004	3.93 [1.76-8.79]	0.001	8.6%	20.9%
1 to <5 y	5,645 (199)	3.62 [1.83-7.18]	<0.001	4.47 [2.18-9.19]	<0.001	62.3%	69.2%
5 to <12 y	1,533 (23)	1.90 [0.91-3.98]	0.088	2.03 [0.96-4.28]	0.064	16.9%	15.1%
**Drug formulation**							
FDC (reference)	4,135 (70)	1	-	-	-	-	-
Co-blistered NFDC	1,256 (21)	1.02 [0.52-2.00]	0.950	1.38 [0.75-2.57]	0.300	13.9%	5.1%
Loose NFDC-25	1,291(70)	3.62 [1.79-7.30]	<0.001	3.51 [2.02-6.12]	<0.001	14.3%	25.8%
Loose NFDC-30^e^							
In Rukara/Kailahun/Kisumu^f^	461 (59)	8.41 [3.24-21.84]	<0.001	7.75 [4.07-14.76]	<0.001	5.1%	26.3%
Rest of the sites	1,952 (45)	1.34 [0.77-2.34]	0.300	1.47 [0.91-2.38]	0.110	21.1%	8.3%
**Treatment supervision**							
Fully supervised (reference)	8,530 (245)	1	-	-	-	-	-
Partially supervised	565 (20)	1.37 [0.45-4.17]	0.580	-	-	-	-
**Parasite clearance**							
Day3 Parasitemia	8,788 (252)	2.17 [0.88-5.35]	0.092	-	-	-	-
**Region**							
Africa (reference)^g^	8,624 (245)	1	-	-	-	-	-
Asia	434 (20)	1.27 [1.83-3.55]	0.700	7.39 [3.45-15.86]	<0.001	4.8%	21.6%
S. America^h^	37 (0)	-	-	-	-		

^a^Number of patients [n] for each variable/levels of factor with number of PCR-confirmed recrudescence [n] by day 28.

^b^Variance of the random effect = 0.22. Adding hemoglobin (AHR = 0.94 [95% CI: 0.88-1.02]; *P* = 0.064), day 3 parasite positivity (AHR = 2.04 [95% CI:0.83-5.00]; *P* = 0.107) to a model containing age, parasitemia, AQ dose, region and formulation led to a non-significant likelihood ratio test, and hence those variables were not kept for multivariable analysis. Although anemia (AHR = 1.35 [95% CI: 1.02-1.78]; *P* = .034) was found to be significant, a large proportion of patients had missing values. Hence, random imputation was performed for anemia, hemoglobin and gametocytemia, which showed that they were not significant in the presence of other variables (Additional file 6: Text S6, Figure 1). To examine the robustness of the parameter estimates, a sensitivity analysis was carried out by removing one study site at a time which showed that the overall coefficient of variation of parameter estimates in the multivariable model was small (all CV <10%) (Additional file 6: Text S6, Table 3).

^c^Overall PAR for model = 92.6%.

^d^Underweight for age defined only in children < 5 years.

^e^Compared to FDC, patients treated with loose NFDC-30 were at higher risk of recrudescence (AHR = 2.89 [95% CI: 1.49-5.59]; *P* = 0.002) when all the sites were combined.

Pairwise comparisons.

Co-blistered NFDC v loose NFDC-25 (AHR = 2.50 [95% CI: 1.18-5.44]; *P* = 0.016).

Co-blistered NFDC v loose NFDC-30 in Rukara/Kailahun/Kisumu (AHR = 5.61 [95% CI: 2.48-12.69]; *P* < 0.001).

Co-blistered NFDC v loose NFDC-30 in rest of the sites (AHR = 1.07 [95% CI: 0.54-2.10]; *P* = 0.850).

Loose NFDC-25 v loose NFDC-30 in Rukara/Kailahun/Kisumu (AHR = 2.21 [95% CI: 1.03-4.71]; *P* = 0.041).

Loose NFDC-25 v loose NFDC-30 in rest of the sites (AHR = 0.42 [95% CI: 0.23-0.77]; *P* = 0.005).

^f^The test for proportional hazards did not hold true for this category. The overall assumption of proportional hazards held true globally and individually for each of the covariates when these three sites were excluded from the model. The coefficients of the remaining model parameters were similar with and without these three sites kept in the model. The assumption of proportionality was tested for each of the studies separately with at least five failures (Additional file 6: Text S6, Table 3) and found to be satisfactory.

^g^Within Africa, there were no differences between East and West Africa: AHR = 1.14 [0.62-2.15]; *P* = 0.690.

^h^Hazards ratio could not be estimated as there were no PCR-confirmed failures in South America.

### Safety parameters

Neutrophil counts were available from five studies (516 treatments), with neutropenia reported in 27 (5.2%) patients at enrolment. In 489 patients with normal neutrophil counts at enrolment, 21.1% (103/489) developed neutropenia (defined as ≤1,200 neutrophils/μl for <12 years and ≤1,500 neutrophils/μl for ≥12 years) within 28 days of follow-up. After adjusting for age and drug formulation, there was no dose-dependent risk of neutropenia (Table 5 in Additional file 6: Text S6).

Data on hemoglobin was available in 33 studies (6,574 treatments), with 57% (3,756/6,574) of the patients anemic at enrolment. Follow-up data were available in 90% (2,557/2,818) of the patients who were not anemic at baseline. In total 23% (590/2,557) developed anemia within 28 days of the follow-up. After adjusting for age category, drug formulation and baseline parasitemia, there was no relationship between drug dose and anemia (Table 5 in Additional file 6: Text S6).

Vomiting within an hour of treatment administration was reported in 12.5% (294/2,351) from seven studies, with the proportion highest in infants <1 year (21.4%, 27/126) and lowest in those 12 years of age or older (4%, 11/278). Data on vomiting within 7 days of treatment were available in 12 studies (3,721 treatments); this occurred in 11% (410/3,721) of the patients. In 12 studies where data for diarrhea were available, 7.6% (290/3,821) reported at least one episode of diarrhea within a week after treatment (Table 7). After controlling for age and drug formulation, the AQ dose was associated with increased risk of diarrhea (adjusted odds ratio, AOR = 1.16 [95% CI: 1.07-1.24]; *P* < 0.001), vomiting (AOR = 1.20 [95% CI: 1.11-1.29]; *P* < 0.001) and vomiting within one hour after treatment (AOR = 1.23 [95% CI: 1.11-1.36]; *P* < 0.001) for every 5 mg/kg increase (Table 5 in Additional file 6: Text S6).

**Table 7:** **Table of adverse events**

	**Neutropenia** ^**a, b**^ **between day 1 and day 28**	**Anemia** ^**a, b**^ **between day 1 and day 28**	**Diarrhea between day 1 and day 7**	**Vomiting** ^**c**^ **between day 1 and day 7**	**Acute drug vomiting**
**AQ dose category (mg/kg)** ^**d**^					
<25	7.0% (5/71)	19.3% (79/410)	4.7% (11/232)	9.3% (24/258)	4.7% (10/214)
25 to <30	20.4% (33/162)	24.1% (190/787)	6.5% (56/857)	9.7% (81/838)	12.9% (80/622)
30 to <35	17.9% (21/117)	21.3% (132/621)	5.8% (55/955)	9.9% (92/933)	11.3% (55/486)
35 to <40	35.3% (30/85)	24.8% (96/387)	7.1% (49/693)	11.3% (74/656)	12.5% (54/433)
40 to <45	30.8% (8/26)	26.9% (58/216)	7.4% (43/580)	13.9% (76/546)	14.7% (62/423)
≥45	21.4% (6/28)	25.7% (35/136)	15.1% (76/504)	12.9% (63/490)	19.1% (33/173)
**Age category**					
<1 y	30.0% (15/50)	49.6% (64/129)	18.4% (52/282)	6.6% (19/287)	21.4% (27/126)
1 to <5 y	17.3% (44/255)	28.0% (437/1,558)	7.4% (189/2,565)	8.7% (228/2,611)	13.9% (230/1,655)
5 to <12 y	13.3% (14/105)	12.4% (50/402)	3.2% (16/505)	15.8% (69/436)	8.9% (26/292)
≥12 y	38.0% (30/79)	8.3% (39/468)	7.0% (33/469)	24.3% (94/387)	4.0% (11/278)
**Overall**	21.1% (103/489)	23.1% (590/2,557)	7.6% (290/3,821)	11.0% (410/3,721)	12.5% (294/2,351)

^a^Presented only for patients without neutropenia/anemia at baseline.

^b^Neutropenia defined as ≤1,200 neutrophils/μl for <12 years and ≤1,500 neutrophils/μl for ≥12 years. Anemia defined as hemoglobin < 10 g/dl.

^c^Excludes acute drug vomiting within an hour of treatment administration.

^d^After adjusting for age category and formulation, AOR = 1.17 [95% CI: 0.95-1.46]; *P* = 0.144 for the risk of neutropenia for every 5 mg/kg increase in AQ dose.

^d^After adjusting for age category and formulation, AOR = 1.16 [95% CI: 1.07-1.24]; *P* < 0.001 for the risk of diarrhea for every 5 mg/kg increase in AQ dose.

^d^After adjusting for age category and formulation, AOR = 1.20 [95% CI: 1.11-1.29]; *P* < 0.001 for the risk of general vomiting for every 5 mg/kg increase in AQ dose.

^d^After adjusting for age category and formulation, AOR = 1.23 [95% CI: 1.11-1.36]; *P* < 0.001 for the risk of acute vomiting for every 5 mg/kg increase in AQ dose.

## Discussion

We collated individual patient data from 43 studies of antimalarial therapy with AS-AQ, including more than 9,000 patients recruited between 1999 and 2012. The data were derived predominantly from studies conducted in sub-Saharan Africa, with a wide range of patient ages, malaria transmission intensities, drug formulations and dosing plans. Three different formulations were included, and all of them were designed to deliver a total target dose of 12 mg/kg of artesunate (AS) over three days; however, the total target dose of amodiaquine (AQ) was 30 mg/kg for FDC and co-blistered NFDC regimens and 25 or 30 mg/kg for loose NFDCs. Overall, the efficacy of AS-AQ was high, but it varied with patient age, formulation and target dose. The efficacy was similar between FDC and co-blistered NFDC, but significantly lower in patients treated with loose NFDCs, and lowest in those treated with an AQ target dose of 25 mg/kg. The efficacy was especially low in infants younger than 1 year treated with all loose NFDCs; below 95% at day 28 and <90% by day 42.

As observed with other ACTs, high baseline parasitemia and young age were significant risk factors for treatment failure, likely explained by the lower immunity in children less than 5 years of age, associated with hyperparasitemia [20,28,29]. However, after controlling for these two confounders, patients treated with the loose NFDC with a target dose of 25 mg/kg were at 3.5-fold greater risk of treatment failure compared to those treated with FDC. In contrast to the variable outcomes among the studies administering loose NFDC, those using the fixed dose combinations reported consistently good AS-AQ efficacy in geographically diverse sites [15,16,18,30-38], with the exception of one study conducted in Myanmar [7].

Several factors could explain the difference in efficacies between the different AS-AQ formulations. The lower efficacy in patients treated with the loose NFDC-25, especially in infants younger than 1 year, is likely to reflect the lower overall dose of AQ administered compared to other patients in this meta-analysis who received a target AQ dose of 30 mg/kg for all other formulations. Moreover, infants <1 year treated with loose NFDC-25 received the lowest AQ dose, which could explain the lower efficacy in this age category. However, due to the limited number of failures in this age group, the dose effect was not apparent in this meta-analysis. The need to split tablets in the loose NFDC regimens could also have contributed to dosing inaccuracy, particularly in young patients, with diminished treatment efficacy in those under-dosed with AQ [39]. Indeed, our results show that even though patients treated with loose NFDC-30 received the same AQ target dose (30 mg/kg) as the patients treated with FDC, the efficacy was still higher in the FDC group. The dosage of the fixed dose combination of AS-AQ was developed using a weight-for-age reference database from malaria endemic countries, to ensure optimal dosing with the pediatric formulation [40]. This allows the FDC prescription to be based either on body weight or age, a notable advantage, as body weight often cannot be assessed easily or accurately in health facilities of many malaria endemic countries. A formulation that can be applied either by weight- or age-based criteria probably increases dosing accuracy, and the availability of different tablet strengths, including a pediatric formulation, obviates the need for tablet splitting, reduces the pill burden and potentially improves adherence [18,41]. The effects on AQ drug concentrations of manufacturer, formulation, age, nutritional status and dosage schedule are currently being evaluated in a separate WWARN amodiaquine PK-PD analysis [42].

In this meta-analysis, AS-AQ efficacy was particularly low in three sites in Rwanda, Sierra Leone and Kenya using loose NFDC with a target AQ dose of 30 mg/kg. Based on the concomitant high failure rates for AQ monotherapy in those sites, AQ resistance was suggested to be a main factor contributing to poor treatment outcomes [11,43,44]. Moreover, patients from Asia were at seven times greater risk of treatment failure compared to patients from Africa, suggesting also that resistance could be responsible for the higher risk of treatment failure in Asia [7,14]. There has been concern that the efficacy of AS-AQ has been compromised by antimalarial resistance to AQ [7-11,44-46]. Parasites carrying the 76 T allele of *pfcrt* are associated with lower susceptibility to AQ, and these parasites are now highly prevalent in most endemic areas [47-52]. Increasing prevalence of the *pfcrt* SVMNT haplotype in some endemic areas has also been associated with AQ use [12-14,53,54]. Resistance has also been invoked to explain the relatively high risks of failure for loose NFDC in some studies [8,9], whereas other studies found adequate efficacy of AS-AQ with this formulation [10,55,56]. Molecular data were not available for this meta-analysis, and associations between AQ resistance markers and treatment outcomes could not be characterised.

Although the primary aim of this analysis was to investigate the effect of AS-AQ dose and formulation on early and late treatment outcomes, we also investigated the effect of these factors on safety outcomes. AQ has previously been associated with neutropenia when taken as a prophylaxis [57] and when used in conjunction with antiretroviral drugs [58]. With limited data, our analysis showed no relationship between the dose of AQ and neutropenia. However, a higher AQ dose was associated with increased risk of gastrointestinal adverse events. A dose-dependent increase in the risk of gastrointestinal adverse events was also reported with artemether-lumefantrine [59].

Our analysis has a number of limitations. Although the search was limited to prospective clinical trials recorded in PubMed, an additional review of clinicaltrials.gov identified that out of the 36 clinical studies registered testing AS-AQ between 2000 and 2012, 28 (78%) had subsequently been published and most of them were included in the meta-analysis. Moreover, our meta-analysis also included seven unpublished clinical trials that were not registered in clinicaltrials.gov. Hence our analysis has captured the majority of published data and constitutes the largest meta-analysis of AS-AQ undertaken. Furthermore there were no apparent differences in patient characteristics and outcomes between the studies included and those which were not available (Table 6 in Additional file 6: Text S6). In addition, the model estimates were robust, as a sensitivity analysis showed that the coefficients of variation for the model parameters were small and the coefficients from the final model were similar to the estimates obtained from bootstrap sampling (Table 3 and Figure 2 in Additional file 6: Text S6). Another limitation of our study was that the FDC trials were mainly conducted in West Africa and those of loose NFDC mainly in East Africa, two regions with reported varied degrees of AQ resistance [14]. Nonetheless, the overall efficacy of the FDC remained consistently high in all regions of Africa and in all age groups. Note that two different FDC formulations with different dosing schemes were included in the analysis; however, it was not possible to assess if that difference could impact on efficacy, as the sample size of one of the formulations was very small. Whilst reassuring, the results of the South American data were limited to one study from Colombia and hence cannot be generalised across the continent. Finally, the information on the actual number of tablets administered, which was used to calculate total drug doses, was available in only 28% (2,570/9,106) of patients. However, when the method of dose calculation was added to the model as a covariate, there was no change in final outcomes.

In summary, this meta-analysis performed with individual patients data highlighted marked heterogeneity in the dosing of AQ between different AS-AQ formulations. These findings also allow differentiation of the impact of formulations from resistance affecting AS-AQ efficacy. The fixed dose combination provided higher efficacy in all age categories, probably reflecting optimal dosing of AQ. AS-AQ FDCs are currently available from five different WHO prequalified manufacturers [60]. In addition to offering improved treatment efficacy, FDCs simplify treatment regimens by reducing the pill burden. A continued concern with all ACTs is impact of resistance to both components on treatment efficacy; thus monitoring of molecular markers associated with resistance to AQ [61,62] and artemisinins [63] is warranted for the combination studied here.

## Additional files

Additional file 1:
**Text S1.** References of all AS-AQ clinical trials, their study designs and dosing schedules. Additional file 2:
**Text S2.** Map of study sites. Additional file 3:
**Text S3.** Transmission classification. Additional file 4:
**Text S4.** WWARN AS-AQ statistical analytical plan. Additional file 5:
**Text S5.** WWARN clinical data and management statistical analytical plan. Additional file 6:
**Text S6.** Additional tables and figures. Additional file 7:
**Text S7.** Authors and contributions.

## Abbreviations

ACT: artemisinin-based combination therapy
AHR: adjusted hazard ratio
AOR: adjusted odds ratio
AQ: amodiaquine
AS: artesunate
AS-AQ: artesunate-amodiaquine
CI: confidence interval
DMSAP: data management and statistical analytical plan
FDC: fixed dose combination
GLURP: glutamate rich protein
Hb: Hemoglobin
IQR: interquartile range
MSP1: merozoite surface protein 1
MSP2: merozoite surface protein 2
NFDC: non-fixed dose combination
OxTREC: Oxford Tropical Research Ethics Committee
PAR: population attributable risk
PCR: polymerase chain reaction
WHO: World Health Organization
WWARN: WorldWide Antimalarial Resistance Network

## References

[1] White NJ (2008). The role of anti-malarial drugs in eliminating malaria. Malar J.

[2] WHO (2014). World Malaria Report 2014.

[3] White N (1999). Antimalarial drug resistance and combination chemotherapy. Philos Trans R Soc L B Biol Sci.

[4] Sinclair D, Zani B, Donegan S, Olliaro P, Garner P (2009). Artemisinin-based combination therapy for treating uncomplicated malaria. Cochrane Database Syst Rev.

[5] Zwang J, Olliaro P, Barennes H, Bonnet M, Brasseur P, Bukirwa H (2009). Efficacy of artesunate-amodiaquine for treating uncomplicated falciparum malaria in sub-Saharan Africa: a multi-centre analysis. Malar J.

[6] Hasugian AR, Purba HL, Kenangalem E, Wuwung RM, Ebsworth EP, Maristela R (2007). Dihydroartemisinin-piperaquine versus artesunate-amodiaquine: superior efficacy and posttreatment prophylaxis against multidrug-resistant Plasmodium falciparum and Plasmodium vivax malaria. Clin Infect Dis.

[7] Smithuis F, Kyaw MK, Phe O, Win T, Aung PP, Oo APP (2010). Effectiveness of five artemisinin combination regimens with or without primaquine in uncomplicated falciparum malaria: an open-label randomised trial. Lancet Infect Dis.

[8] Adjuik M, Agnamey P, Babiker A, Borrmann S, Brasseur P, Cisse M (2002). Amodiaquine-artesunate versus amodiaquine for uncomplicated Plasmodium falciparum malaria in African children: a randomised, multicentre trial. Lancet.

[9] Mutabingwa TK, Anthony D, Heller A, Hallett R, Ahmed J, Drakeley C (2005). Amodiaquine alone, amodiaquine + sulfadoxine-pyrimethamine, amodiaquine + artesunate, and artemether-lumefantrine for outpatient treatment of malaria in Tanzanian children: a four-arm randomised effectiveness trial. Lancet.

[10] Bonnet M, Broek I, van Herp M, Urrutia PP, van Overmeir C, Kyomuhendo J (2009). Varying efficacy of artesunate + amodiaquine and artesunate + sulphadoxine-pyrimethamine for the treatment of uncomplicated falciparum malaria in the Democratic Republic of Congo: a report of two in-vivo studies. Malar J.

[11] Rwagacondo CE, Karema C, Mugisha V, Erhart A, Dujardin JC, Van Overmeir C (2004). Is amodiaquine failing in Rwanda? Efficacy of amodiaquine alone and combined with artesunate in children with uncomplicated malaria. Trop Med Int Health.

[12] Alifrangis M, Dalgaard MB, Lusingu JP, Vestergaard LS, Staalsoe T, Jensen AT (2006). Occurrence of the Southeast Asian/South American SVMNT haplotype of the chloroquine-resistance transporter gene in Plasmodium falciparum in Tanzania. J Infect Dis.

[13] Sa JM, Twu O (2010). Protecting the malaria drug arsenal: halting the rise and spread of amodiaquine resistance by monitoring the PfCRT SVMNT type. Malar J.

[14] Sa JM, Twu O, Hayton K, Reyes S, Fay MP, Ringwald P (2009). Geographic patterns of Plasmodium falciparum drug resistance distinguished by differential responses to amodiaquine and chloroquine. Proc Natl Acad Sci U S A.

[15] The Four Artemisinin-Based Combinations (4ABC) Study Group. A head-to-head comparison of four artemisinin-based combinations for treating uncomplicated malaria in African children: a randomized trial. PLoS Med. 2011;8:e1001119. 10.1371/journal.pmed.1001119 PMC3210754 22087077

[16] Thanh NX, Trung TN, Phong NC, Quang HH, Dai B, Shanks GD (2012). The efficacy and tolerability of artemisinin-piperaquine (Artequick(R)) versus artesunate-amodiaquine (Coarsucam) for the treatment of uncomplicated Plasmodium falciparum malaria in south-central Vietnam. Malar J.

[17] Brasseur P, Agnamey P, Gaye O, Cisse M, Badiane M, Vaillant M (2009). Dosing accuracy of artesunate and amodiaquine as treatment for falciparum malaria in Casamance, Senegal. Trop Med Int Health.

[18] Sirima SB, Tiono AB, Gansane A, Diarra A, Ouedraogo A, Konate AT (2009). The efficacy and safety of a new fixed-dose combination of amodiaquine and artesunate in young African children with acute uncomplicated Plasmodium falciparum. Malar J.

[19] Clinical trials review. http://www.wwarn.org/tools-resources/literature-reviews/wwarn-clinical-trials-publication-library/methodology.

[20] WWARN DP Study Group TWARN (WWARN) DS Group: The effect of dosing regimens on the antimalarial efficacy of dihydroartemisinin-piperaquine: a pooled analysis of individual patient data. PLoS Med. 2013, 10:e1001564. doi:10.1371/journal.pmed.1001564. 10.1371/journal.pmed.1001564 PMC3848996 24311989

[21] Gething PW, Patil AP, Smith DL, Guerra CA, Elyazar IR, Johnston GL (2011). A new world malaria map: Plasmodium falciparum endemicity in 2010. Malar J.

[22] WWARN: Statistical Analysis Plan, AS-AQ Dose Impact Study Group. Version 1.9. WordlWide Antimalarial Resistance Network, Oxford, 2012.

[23] WWARN. Clinical Module: Data Management and Statistical Analysis Plan. Version 1.2. WorldWide Antimalarial Resistance Network, Oxford, 2012.

[24] Glidden DV, Vittinghoff E (2004). Modelling clustered survival data from multicentre clinical trials. Stat Med.

[25] Munda M, Legrand C (2014). Adjusting for centre heterogeneity in multicentre clinical trials with a time-to-event outcome. Pharm Stat.

[26] Levin ML (1953). The occurrence of lung cancer in man. Acta Unio Int Contra Cancrum.

[27] Guidelines for the treatment of malaria, 2nd ed. http://www.who.int/malaria/publications/atoz/9789241547925/en/

[28] Price R, Luxemburger C, van Vugt M, Nosten F, Kham A, Simpson J (1998). Artesunate and mefloquine in the treatment of uncomplicated multidrug-resistant hyperparasitaemic falciparum malaria. Trans R Soc Trop Med Hyg.

[29] Nacher M, Carrara VI, Ashley E, McGready R, Hutagalung R, Nguen JV (2004). Seasonal variation in hyperparasitaemia and gametocyte carriage in patients with Plasmodium falciparum malaria on the Thai-Burmese border. Trans R Soc Trop Med Hyg.

[30] Ndiaye JL, Randrianarivelojosia M, Sagara I, Brasseur P, Ndiaye I, Faye B (2009). Randomized, multicentre assessment of the efficacy and safety of ASAQ - a fixed-dose artesunate-amodiaquine combination therapy in the treatment of uncomplicated Plasmodium falciparum malaria. Malar J.

[31] Sinou V, Malaika LT, Taudon N, Lwango R, Alegre SS, Bertaux L (2009). Pharmacokinetics and pharmacodynamics of a new ACT formulation: Artesunate/Amodiaquine (TRIMALACT) following oral administration in African malaria patients. Eur J Drug Metab Pharmacokinet.

[32] Ndiaye JL, Faye B, Gueye A, Tine R, Ndiaye D, Tchania C (2011). Repeated treatment of recurrent uncomplicated Plasmodium falciparum malaria in Senegal with fixed-dose artesunate plus amodiaquine versus fixed-dose artemether plus lumefantrine: a randomized, open-label trial. Malar J.

[33] De la Hoz RF, Porras Ramirez A, Rico Mendoza A, Cordoba F, Rojas DP (2012). Artesunate + amodiaquine versus artemether-lumefantrine for the treatment of uncomplicated Plasmodium falciparum malaria in the Colombian Pacific region: a noninferiority trial. Rev Soc Bras Med Trop.

[34] Espie E, Lima A, Atua B, Dhorda M, Flevaud L, Sompwe EM (2012). Efficacy of fixed-dose combination artesunate-amodiaquine versus artemether-lumefantrine for uncomplicated childhood Plasmodium falciparum malaria in Democratic Republic of Congo: a randomized non-inferiority trial. Malar J.

[35] Faye B, Kuete T, Kiki-Barro CP, Tine RC, Nkoa T, Ndiaye JL (2012). Multicentre study evaluating the non-inferiority of the new paediatric formulation of artesunate/amodiaquine versus artemether/lumefantrine for the management of uncomplicated Plasmodium falciparum malaria in children in Cameroon, Ivory Coast and Senegal. Malar J.

[36] Anvikar AR, Sharma B, Shahi BH, Tyagi PK, Bose TK, Sharma SK (2012). Artesunate-amodiaquine fixed dose combination for the treatment of Plasmodium falciparum malaria in India. Malar J.

[37] Ndounga M, Mayengue PI, Casimiro PN, Loumouamou D, Basco LK, Ntoumi F (2013). Artesunate-amodiaquine efficacy in Congolese children with acute uncomplicated falciparum malaria in Brazzaville. Malar J.

[38] Schramm B, Valeh P, Baudin E, Mazinda CS, Smith R, Pinoges L (2013). Efficacy of artesunate-amodiaquine and artemether-lumefantrine fixed-dose combinations for the treatment of uncomplicated Plasmodium falciparum malaria among children aged six to 59 months in Nimba County, Liberia: an open-label randomized non-inferiority. Malar J.

[39] Elliott I, Mayxay M, Yeuichaixong S, Lee SJ, Newton PN (2014). The practice and clinical implications of tablet splitting in international health. Trop Med Int Health.

[40] Taylor W, Terlouw DJ, Olliaro PL, White NJ, Brasseur P, ter Kuile FO (2006). Use of weight-for-age-data to optimize tablet strength and dosing regimens for a new fixed-dose artesunate-amodiaquine combination for treating falciparum malaria. Bull World Health Organ.

[41] Connor J, Rafter N, Rodgers A (2004). Do fixed-dose combination pills or unit-of-use packaging improve adherence? A systematic review. Bull World Health Organ.

[42] Amodiaquine PK/PD Study Group. http://www.wwarn.org/working-together/study-groups/amodiaquine-pkpd-study-group

[43] Eyase FL, Akala HM, Ingasia L, Cheruiyot A, Omondi A, Okudo C (2013). The role of Pfmdr1 and Pfcrt in changing chloroquine, amodiaquine, mefloquine and lumefantrine susceptibility in western-Kenya P. falciparum samples during 2008-2011. PLoS One.

[44] Grandesso F, Hagerman A, Kamara S, Lam E, Checchi F, Balkan S (2006). Low efficacy of the combination artesunate plus amodiaquine for uncomplicated falciparum malaria among children under 5 years in Kailahun, Sierra Leone. Trop Med Int Health.

[45] Martensson A, Stromberg J, Sisowath C, Msellem MI, Gil JP, Montgomery SM (2005). Efficacy of artesunate plus amodiaquine versus that of artemether-lumefantrine for the treatment of uncomplicated childhood Plasmodium falciparum malaria in Zanzibar, Tanzania. Clin Infect Dis.

[46] Thwing JI, Odero CO, Odhiambo FO, Otieno KO, Kariuki S, Ord R (2009). In-vivo efficacy of amodiaquine-artesunate in children with uncomplicated Plasmodium falciparum malaria in western Kenya. Trop Med Int Health.

[47] Ochong EO, van den Broek IV, Keus K, Nzila A (2003). Short report: association between chloroquine and amodiaquine resistance and allelic variation in the Plasmodium falciparum multiple drug resistance 1 gene and the chloroquine resistance transporter gene in isolates from the upper Nile in southern Sudan. Am J Trop Med Hyg.

[48] Happi CT, Gbotosho GO, Folarin OA, Bolaji OM, Sowunmi A, Kyle DE (2006). Association between mutations in Plasmodium falciparum chloroquine resistance transporter and P. falciparum multidrug resistance 1 genes and in vivo amodiaquine resistance in P. falciparum malaria-infected children in Nigeria. Am J Trop Med Hyg.

[49] Echeverry DF, Holmgren G, Murillo C, Higuita JC, Bjorkman A, Gil JP (2007). Short report: polymorphisms in the pfcrt and pfmdr1 genes of Plasmodium falciparum and in vitro susceptibility to amodiaquine and desethylamodiaquine. Am J Trop Med Hyg.

[50] Nsobya SL, Dokomajilar C, Joloba M, Dorsey G, Rosenthal PJ (2007). Resistance-mediating Plasmodium falciparum pfcrt and pfmdr1 alleles after treatment with artesunate-amodiaquine in Uganda. Antimicrob Agents Chemother.

[51] Holmgren G, Hamrin J, Svard J, Martensson A, Gil JP, Bjorkman A (2007). Selection of pfmdr1 mutations after amodiaquine monotherapy and amodiaquine plus artemisinin combination therapy in East Africa. Infect Genet Evol.

[52] Danquah I, Coulibaly B, Meissner P, Petruschke I, Muller O, Mockenhaupt FP (2010). Selection of pfmdr1 and pfcrt alleles in amodiaquine treatment failure in north-western Burkina Faso. Acta Trop.

[53] Dittrich S, Alifrangis M, Stohrer JM, Thongpaseuth V, Vanisaveth V, Phetsouvanh R (2005). Falciparum malaria in the north of Laos: the occurrence and implications of the Plasmodium falciparum chloroquine resistance transporter (pfcrt) gene haplotype SVMNT. Trop Med Int Health.

[54] Beshir K, Sutherland CJ, Merinopoulos I, Durrani N, Leslie T, Rowland M (2010). Amodiaquine resistance in Plasmodium falciparum malaria in Afghanistan is associated with the pfcrt SVMNT allele at codons 72 to 76. Antimicrob Agents Chemother.

[55] Bukirwa H, Yeka A, Kamya MR, Talisuna A, Banek K, Bakyaita N (2006). Artemisinin combination therapies for treatment of uncomplicated malaria in Uganda. PLoS Clin Trials.

[56] Thanh NX, Trung TN, Phong NC, Thien NX, Dai B, Shanks GD (2009). Open label randomized comparison of dihydroartemisinin-piperaquine and artesunate-amodiaquine for the treatment of uncomplicated Plasmodium falciparum malaria in central Vietnam. Trop Med Int Heal.

[57] Hatton CS, Peto TE, Bunch C, Pasvol G, Russell SJ, Singer CR (1986). Frequency of severe neutropenia associated with amodiaquine prophylaxis against malaria. Lancet.

[58] Gasasira AF, Kamya MR, Achan J, Mebrahtu T, Kalyango JN, Ruel T (2008). High risk of neutropenia in HIV-infected children following treatment with artesunate plus amodiaquine for uncomplicated malaria in Uganda. Clin Infect Dis.

[59] WWARN AL Study Group TWARNA Dose Impact S Group. The effect of dose on the antimalarial efficacy of artemether-lumefantrine: a pooled analysis of individual patient data. Lancet Infect Dis. 2015; In press. 10.1016/S1473-3099(15)70024-1 PMC4632191 25788162

[60] WHO List of Prequalified Medicinal Products. http://apps.who.int/prequal/query/ProductRegistry.aspx.

[61] Ecker A, Lehane AM, Clain J, Fidock DA (2012). PfCRT and its role in antimalarial drug resistance. Trends Parasitol.

[62] Rosenthal PJ (2013). The interplay between drug resistance and fitness in malaria parasites. Mol Microbiol.

[63] Ariey F, Witkowski B, Amaratunga C, Beghain J, Langlois A-C, Khim N (2014). A molecular marker of artemisinin-resistant Plasmodium falciparum malaria. Nature.

[64] Barennes H, Nagot N, Valea I, Koussoube-Balima T, Ouedraogo A, Sanou T (2004). A randomized trial of amodiaquine and artesunate alone and in combination for the treatment of uncomplicated falciparum malaria in children from Burkina Faso. Trop Med Int Heal.

[65] Bonnet M, Roper C, Félix M, Coulibaly L, Kankolongo GM, Guthmann JP (2007). Efficacy of antimalarial treatment in Guinea: in vivo study of two artemisinin combination therapies in Dabola and molecular markers of resistance to sulphadoxine-pyrimethamine in N’Zérékoré. Malar J.

[66] Bukirwa H, Critchley J (2006). Sulfadoxine-pyrimethamine plus artesunate versus sulfadoxine-pyrimethamine plus amodiaquine for treating uncomplicated malaria. Cochrane Database Syst Rev.

[67] Dorsey G, Staedke S, Clark TD, Njama-Meya D, Nzarubara B, Maiteki-Sebuguzi C (2007). Combination therapy for uncomplicated falciparum malaria in Ugandan children: a randomized trial. JAMA.

[68] Faucher JF, Aubouy A, Adeothy A, Cottrell G, Doritchamou J, Gourmel B (2009). Comparison of sulfadoxine-pyrimethamine, unsupervised artemether-lumefantrine, and unsupervised artesunate-amodiaquine fixed-dose formulation for uncomplicated plasmodium falciparum malaria in Benin: a randomized effectiveness noninferiority trial. J Infect Dis.

[69] Faye B, Offianan AT, Ndiaye JL, Tine RC, Toure W, Djoman K (2010). Efficacy and tolerability of artesunate-amodiaquine (Camoquin plus) versus artemether-lumefantrine (Coartem) against uncomplicated Plasmodium falciparum malaria: multisite trial in Senegal and Ivory Coast. Trop Med Int Health.

[70] Guthmann JP, Ampuero J, Fortes F, van Overmeir C, Gaboulaud V, Tobback S (2005). Antimalarial efficacy of chloroquine, amodiaquine, sulfadoxine-pyrimethamine, and the combinations of amodiaquine + artesunate and sulfadoxine-pyrimethamine + artesunate in Huambo and Bie provinces, central Angola. Trans R Soc Trop Med Hyg.

[71] Guthmann JP, Cohuet S, Rigutto C, Fortes F, Saraiva N, Kiguli J (2006). High efficacy of two artemisinin-based combinations (artesunate + amodiaquine and artemether + lumefantrine) in Caala, Central Angola. Am J Trop Med Hyg.

[72] Hamour S, Melaku Y, Keus K, Wambugu J, Atkin S, Montgomery J (2005). Malaria in the Nuba Mountains of Sudan: baseline genotypic resistance and efficacy of the artesunate plus sulfadoxine-pyrimethamine and artesunate plus amodiaquine combinations. Trans R Soc Trop Med Hyg.

[73] Jullien V, Ogutu B, Juma E, Carn G, Obonyo C, Kiechel JR (2010). Population pharmacokinetics and pharmacodynamic considerations of amodiaquine and desethylamodiaquine in Kenyan adults with uncomplicated malaria receiving artesunate-amodiaquine combination therapy. Antimicrob Agents Chemother.

[74] Karema C, Fanello CI, van Overmeir C, van Geertruyden JP, van Doren W, Ngamije D (2006). Safety and efficacy of dihydroartemisinin/piperaquine (Artekin) for the treatment of uncomplicated Plasmodium falciparum malaria in Rwandan children. Trans R Soc Trop Med Hyg.

[75] Kayentao K, Maiga H, Newman RD, McMorrow ML, Hoppe A, Yattara O (2009). Artemisinin-based combinations versus amodiaquine plus sulphadoxine-pyrimethamine for the treatment of uncomplicated malaria in Faladje, Mali. Malar J.

[76] Ménard D, Ratsimbasoa A, Randrianarivelojosia M, Rabarijaona L-P, Raharimalala L, Domarle O (2008). Assessment of the efficacy of antimalarial drugs recommended by the National Malaria Control Programme in Madagascar: up-dated baseline data from randomized and multi-site clinical trials. Malar J.

[77] Osorio L, Gonzalez I, Olliaro P, Taylor WR (2007). Artemisinin-based combination therapy for uncomplicated Plasmodium falciparum malaria in Colombia. Malar J.

[78] Sagara I, Fofana B, Gaudart J, Sidibe B, Togo A, Toure S (2012). Repeated artemisinin-based combination therapies in a malaria hyperendemic area of Mali: efficacy, safety, and public health impact. Am J Trop Med Hyg.

[79] Staedke SG, Mpimbaza A, Kamya MR, Nzarubara BK, Dorsey G, Rosenthal PJ (2004). Combination treatments for uncomplicated falciparum malaria in Kampala, Uganda: randomised clinical trial. Lancet.

[80] Swarthout TD, van den Broek IV, Kayembe G, Montgomery J, Pota H, Roper C (2006). Artesunate + amodiaquine and artesunate + sulphadoxine-pyrimethamine for treatment of uncomplicated malaria in Democratic Republic of Congo: a clinical trial with determination of sulphadoxine and pyrimethamine-resistant haplotypes. Trop Med Int Health.

[81] Van den Broek I, Kitz C, Al Attas S, Libama F, Balasegaram M, Guthmann J-P (2006). Efficacy of three artemisinin combination therapies for the treatment of uncomplicated Plasmodium falciparum malaria in the Republic of Congo. Malar J.

[82] Yeka A, Banek K, Bakyaita N, Staedke SG, Kamya MR, Talisuna A (2005). Artemisinin versus nonartemisinin combination therapy for uncomplicated malaria: randomized clinical trials from four sites in Uganda. PLoS Med.

