# Supplementary material for: The effect of dosing strategies on the therapeutic efficacy of artesunate-amodiaquine for uncomplicated malaria: a meta-analysis of individual patient data
Source: BMC Med. 2015 Mar 31;13:66. doi: 10.1186/s12916-015-0301-z (PMC4411752; doi:10.1186/s12916-015-0301-z)
Supplement: Additional file 2: — Text S2. Map of study sites. [file 12916_2015_301_MOESM2_ESM.pdf]

Map showing study sites of published AS-AQ clinical studies in Africa, Asia/Oceania and South America

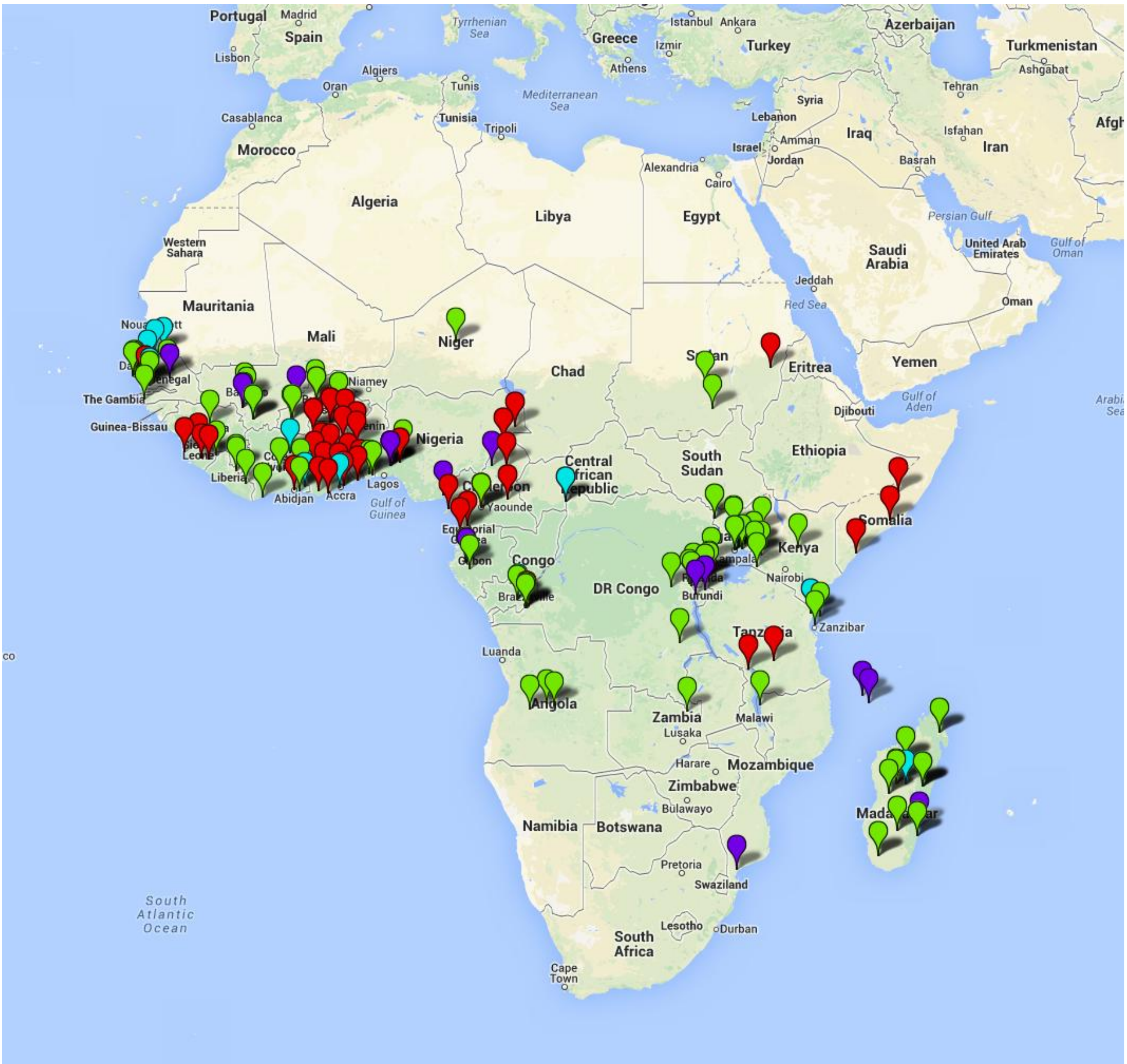

- Studies included in the pooled analysis
- Studies excluded from the pooled analysis
- Studies not available for the pooled analysis
- Studies not targeted for the pooled analysis

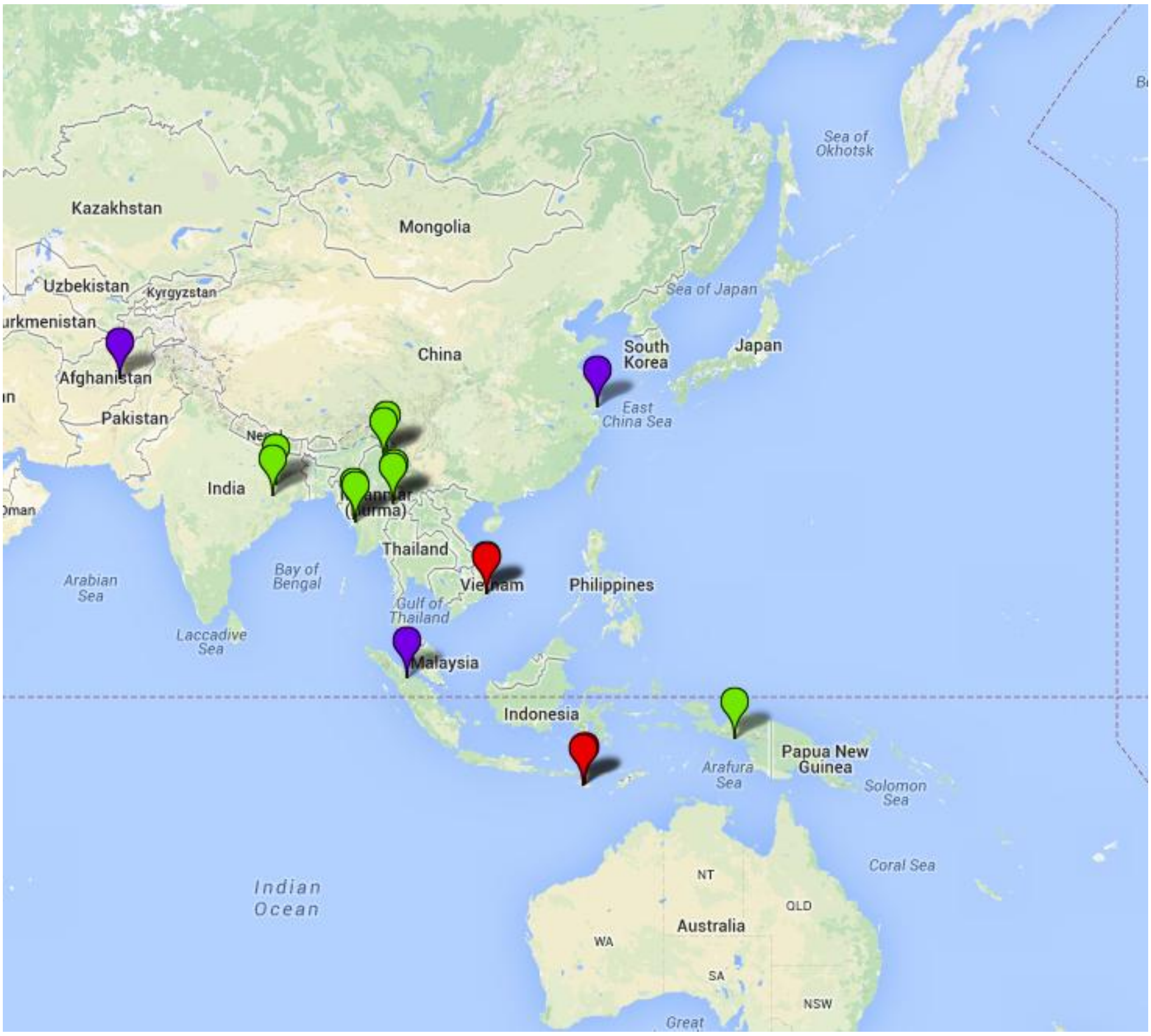

- Studies included in the pooled analysis
- Studies excluded from the pooled analysis
- Studies not available for the pooled analysis
- Studies not targeted for the pooled analysis

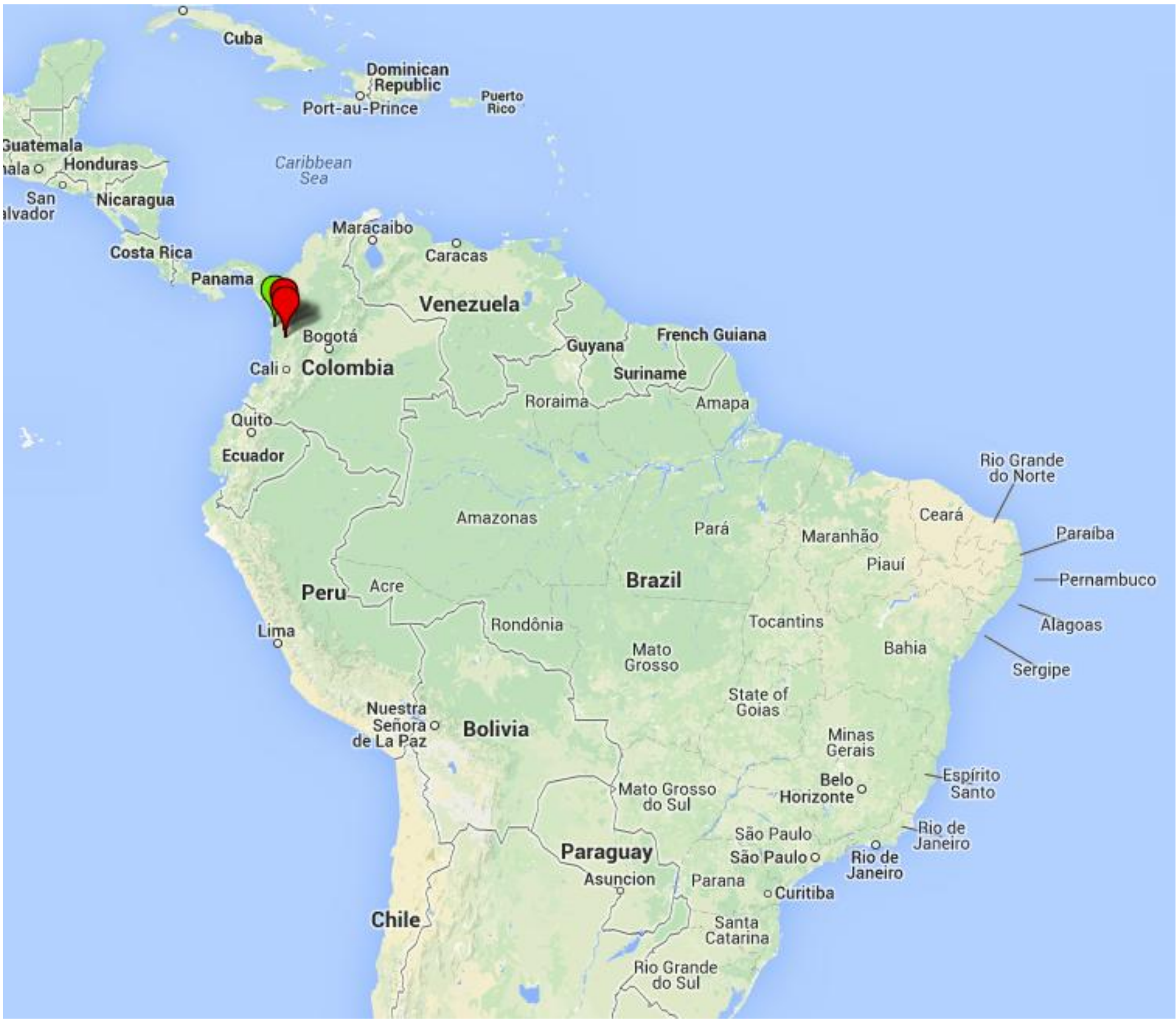

- Studies included in the pooled analysis
- Studies excluded from the pooled analysis
- Studies not available for the pooled analysis
- Studies not targeted for the pooled analysis
