# Supplementary material for: The effect of dosing strategies on the therapeutic efficacy of artesunate-amodiaquine for uncomplicated malaria: a meta-analysis of individual patient data
Source: BMC Med. 2015 Mar 31;13:66. doi: 10.1186/s12916-015-0301-z (PMC4411752; doi:10.1186/s12916-015-0301-z)
Supplement: Additional file 4: — Text S4. WWARN AS-AQ statistical analytical plan. [file 12916_2015_301_MOESM4_ESM.pdf]

Statistical Analysis Plan
AS-AQ Dose Impact Study Group
Version 1.9

WorldWide Antimalarial Resistance Network (WWARN)

Suggested citation: Statistical Analysis Plan, AS-AQ Dose Imapact Study Group: Investigating the effect of mg/kg drug dosage on the clinical outcome

Version History

Version number	Revision(s) & reason for amendment	Release date
**v1.9**		**22.03.12**

WorldWide Antimalarial Resistance Network (WWARN)
www.wwarn.org

Contents

1. Introduction and Rationale 3
2. Outline of Statistical Analysis 4
3. Statistical Methodology 6
4. Tools 7
5. References 8
6. Annex 9

1. Introduction and Rationale

The efficacy of Artemisinin Combination Therapies (ACTs) is influenced by both the artemisinin derivative and the partner drug. Their role is to cure patients but also to prevent the survival and spread of artemisinin resistant strains of Plasmodium. Three of the most common partner drugs currently prescribed for the treatment of uncomplicated malaria are lumefantrine (LUM), piperaquine (PIP), and amodiaquine (AQ). The dosage of partner drugs must be sufficient to ensure that blood concentrations exceed the minimum inhibitory concentration of the parasite until all parasites are been killed. Although target doses are usually given as a total mg/kg over three days, in practice manufacturers' recommendations are often pragmatic and based upon weight "banding". This approach inevitably results in some patients at the margins of having either lower or higher dosages.

Young children are particularly vulnerable to extreme total dosages especially when drug administration is based on tablets (or fractions thereof) rather paediatric formulations or suspensions. The problem is further confounded when dosing is recommended according to age bands rather than actual body weight. Under-dosing has been suggested to play a role in the development of resistance (Terlouw *et. al.*, 2003). Preliminary modelling of dosing strategies according to known weight for age demographics in malaria patients, suggests a wide range of mg/kg dosing is used that may impact significantly on treatment efficacy and possibly safety.

Aim of the study

To determine the mg/kg dosing range of the ACT partner drug Amodiaquine, adopted in clinical trials and investigate the effects of mg/kg dosing on clinical outcome.

Eligibility criteria for inclusion in pooled analysis

A study will be deemed eligible for the purpose of this analysis if they meet the following criteria:

- Prospective clinical efficacy studies of *P. falciparum* (either alone or mixed infections).
- Treatment with **Artesunate-Amodiaquine** with a minimum of 28 days of follow up.
- Data is available on exact dosage of amodiaquine, received by patients (or the dosing table used in the study).
- PCR genotyping results to distinguish between recrudescence and new infections.

Desirable criteria –but not required for inclusion

- Drug manufacturer
- Drug supervision
- Co-adminstration with fat
- Hb or hct during follow up
- Malnutrition as gauged by weight and age +/- height
- Background endemicitiy or transmission prevelance at the time of conducting the study

Exclusion Criteria

- Study in pregnant women
- Study in healthy volunteers
- Study with no intermediate follow-up (e.g. followed up on days 1, 2 and 3 and then on day28 only)

The data sets uploaded to the WWARN repository will be standardized using the WWARN Data Management and Statistical Analysis Plans (DMSAP v1.1[1]) for clinical data and pooled into a single database of quality-assured individual patient data. Data will remain the property of the individual donor(s) and publication will be in accordance with an agreed publication plan (see publication policy document[2]).

2. Outline of Statistical Analysis

Statistical analyses will be take into account the differences in different formulations of this treatment regimen:

- Amodiaquine plus artesunate
    - Fixed Dose Combinations (FDC)
    - Non-Fixed Dose (nFDC)
        - In Loose formulation
        - In Co-Blisters formulation

Baseline characteristics of the studies included in the analysis

An overall summary (overall study profile) of all the studies uploaded to WWARN repository will be presented and studies in which individual mg/kg dosing is available will be ascertained.

Baseline characteristics of studies eligible for the purpose of this project will be presented including information on transmission intensity, age, sex, weight, underweight for age (based on weight for age scores), baseline parasitaemia, species (% of mixed infections), gametocytes on presentation, past history of malaria (if available), haemoglobin, level of supervision of drug intake (full or partial). Haematocrit will be converted to haemoglobin using the following relationship:

$$Hematocrit\ (ht) = 5.62 + 2.60 * Haemoglobin\ (hb)$$ (Lee *et al.*, 2008)

[1] http://www.wwarn.org/partnerships/data/methodology/clinical
[2] http://www.wwarn.org/sites/default/files/PublicationPolicy.pdf

Study locations will be categorised into three strata according to known epidemiology: low, moderate and high transmission settings and also according to region (Africa, Asia, and S. America). Transmission settings will be defined based on observed reinfection rate and prevalence estimates obtained from Malarial Atlas project (MAP). Transmission intensity will be classified as low, moderate or high.

Diversity in dosing strategies

Descriptive statistics for different methods of dosing strategies (age based, weight based) will be calculated. Summary statistics of dosing strategies used in children (e.g. % studies using quarter/half tablets, % dissolving tablets, or paediatric formulation) will be reported when such information is available.

The mg/kg distribution of artemisinin and AQ will be presented using box plots or histograms together with their descriptive statistics.

Scatter plot of mg/kg dosing (Y axis) and age (X axis).

Scatter plot of mg/kg dosing (Y axis) and weight (X axis).

Efficacy endpoints

Primary: PCR adjusted risk of *P. falciparum* recrudesence

Secondary: PCR adjusted risk of *P. falciparum* reinfection
PCR unadjusted risk of *P. falciparum* recurrence
Early parasitological response

Tertiary: Gastrointenstinal adverse events(diarrhea, vomiting and dose vomiting)
Anaemia and neutropeania during the 28 days follow-up

All the primary and secondary endpoints on interest will go through the same analysis as outlined below. Analysis of tertiary endpoint will be carried out provided enough data is present; else, only summary statistics will be reported.

For the cumulative risk of recurrence (*P. falciparum* adjusted, *P. falciparum* unadjusted, and *P. vivax*), survival and Cox regression analysis will be used (section 3). Definitions of status and censorship are detailed on page 14 of the Clinical Module DMSAP v1.1. In addition, the median time to presentation with recurrent infection will be calculated.

For the parasite clearance, the proportions of patients cleared at day 1, 2 and 3 will be assessed. Definitions are detailed on page 15 of the Clinical Module DMSAP v1.1. Univariate risk factors will be assessed by logistic regression for each day adjusting for the study effect. Multivariable analysis will then be carried out using logistic regression.

For the tertiary endpoints, proportion of patients reporting adverse events will be summarised for different age categories and dose-categories for amodiaquine (<25 mg/kg, 25 to <30 mg/kg, 30 to

<35 mg/kg, 40 to <45 mg/kg and ≥ 45 mg/kg). Univariable and multivariable analysis for the risk of adverse events will then be carried out using mixed effects logistic regression with study sites fitted as a random effect.

3. Statistical Methodology

Descriptive statistics

Normality of the distribution of the drug dosages (mg/kg total) will be checked using a Q-Q plot. The p-value from Shapiro-Wilk's normality test will be reported.

The mean, 10th, 25th, 75th, 90th percentile and the median mg/kg dose will be calculated for each dosing group (under/below as defined in Section 2.3.4). The distribution of the drug dosages will be presented graphically (histogram, box plots).

Patients will be categorised into four age groups <1 year, ≥1-<5 years, ≥5-<12 years and ≥12 years. The summary statistics will be further broken down by gender and age category.

Survival analysis

PCR Adjusted and Unadjusted outcomes (ref: DMSAP v1.1 for definitions) obtained using WWARN standardised outputs will be used to compute the Kaplan-Meier (K-M) estimates for the two dosing groups. The K-M estimates will be presented graphically together with the associated tables. Log rank test stratified by study will be performed at 5% level of significance to test if the K-M profiles are significantly different from each other.

Frailty analysis will be carried out (specifying study effect as random) to adjust for the differences between the study sites (Glidden and Vittinghoff, 2004).

Model selection for determinants

Univariable analysis of confounding factors (with study sites fitted as a random effect) associated with the primary and secondary endpoint of interests will be conducted. Model building will be carried out first by investigating if any of the available variables (Annex A.1) are related to the treatment outcome using Cox's regression model. Any variables which are significant in the univariable analysis at 10% level of significance will be kept for multivariable analysis and model selection. During model building, known confounders (age-category, baseline parasitaemia, drug formulations and region) will be always be kept into the model even if they are statistically non-significant.

Model with known confounders will be fitted first (baseline model). Variables and covariates will then be added to the baseline model and the Likelihood Ratio Test (LRT) i.e. changes in log likelihood $(-2\ Log\hat{L})$ will be compared (for nested models) to identify the variables which results in a significant reduction in $-2\ Log\hat{L}$ at 10% level of significance (equivalent to using the model deviance). Akaike's Information Criterion (AIC) will be used to compare competing non-nested models; models with smaller AIC will be preferred. The final model will then be used to estimate

the hazards ratio (HR) for each of the covariates and associated 95% Confidence Interval will be reported.

Population Attributable Risk (PAR) associated with each of the covairtaes found to be significant in the final multivariable model would be estimated using:

$$[\text{prevalence} \times (\text{AHR-1})]/\ \{1+[\text{prevalence} \times (\text{AHR-1})]\}$$

Where prevalence is the proportion of the patients with the level of covariates in the study population and AHR is the adjusted hazard ratio from the final multivariable model. The overall PAR (for a combination of factors), which is non-additive, will be calculated as 1-[(1-PAR1) × (1-PAR2) ×...× (1-PARn)].

Residual Analysis

Cox-Snell's residual and Martingale's residual will be examined to determine the appropriateness of model fit. Schoenfeld residuals against transformed time will be used to determine if the assumptions of PH are reasonable. Any systematic departures from horizontal lines are indicative of non-proportional hazards (Schoenfeld, 1982) and any violation of this assumption will be reported.

Sensitivity Analysis

The robustness of the model coefficients will be explored by exploring the effect of a study site on on the parameter estimates. The coefficient of variation (CV, %) will be reported for each of the model parameters when each study site is removed one at a time.

In addition, a bootstrap analysis will be be performed by taking a random sample of size n (where n= sample size of the final model) and this will be repeated 1000 times. The distribution of the parameter estimates will be presented as a supplementary file.

Finding the optimal cut points (when dose is a significant predictor)

The suitability of Receiving Operating Characteristics Curves (ROCs) for finding the optimal cutpoints for mkg/kg drug dosage which best differentiates the PCR adjusted failure will be explored. The optimal cutoff point will be defined as the maximum value of Youden's Index (*J*), which is sensitivity+ specificity-1 (Price *et al.*, 2007). The use of Logrank statistics to define the breakpoint will be explored. The value of the drug dosage which maximizes the logrank statistics will be identified as the optimal threshold (Contal and O' Quigley, 1999). Alternative methods for determining the optimal cutoff will also be explored (e.g. data driven methods such as percentiles). Outcome oriented approaches will also be explored.

4. Tools

All statistical analyses will be carried out using R 2.14.0 released on 2011-10-31 by The R Foundation for Statistical Computing. However, when equivalent statistical methods are applied, changing the use of statistical software does not require amendment of this SAP.

5. References

Collett, D. (2003) Modelling Survival data in Medical Research. Second edition. Boca Raton: Chapman and Hall/CRC.

Contal, C., O'Quigley, J. "An application of changepoint methods in studying the effect of age on survival in breast cancer," Computational Statistics and Data Analysis, 1999; 30, 253 - 270.
Stable URL: http://www.sciencedirect.com/science/article/pii/S0167947398000966
Accessed: 15/01/2012

Glidden, D.V., and Vittinghoff, E. "Modelling clustered survival data from multicentre clinical trials". Statistics In Medicine.Statist. Med. 2004; 23:369–388 (DOI: 10.1002/sim.1599)
Stable URL: http://onlinelibrary.wiley.com/doi/10.1002/sim.1599/pdf
Accessed: 27/03/2012

Mandrekar JN , Mandrekar SJ , Cha SS . Cutpoint determination methods in survival analysis using SAS®. Proceedings of the 28th SAS Users Group International Conference (SUGI), 2003; 261-28.
Stable URL: http://www2.sas.com/proceedings/sugi28/261-28.pdf
Accessed: 15/01/2012

Lee, S.J, Stepniewska ,K., Anstey, N., Ashley, E., Barnes, K., Binh, T.Q., D'Alessandro, A., Day, N.P.J., de Vries, PJ., Dorsey,G., Guthmann, JP., Mayxa, M., Newton,P., Nosten, F., Olliaro,P., Osario,L., Pinoges,L., Price,R., Rowland,M., Smithuis,F., Taylor,R., White,N.J. (2008). "The relationship between the haemoglobin concentration and the haematocrit in Plasmodium falciparum malaria". Malaria Journal 2008, 7:149 doi: 10.1186/1475-2875-7-149.
Stable URL: http://www.malariajournal.com/content/7/1/149
Accessed: 21/03/2012

Price, R.N., Hasugian,A.R., Ratcliff,A., Siswantoro,H., Purba, H.L.E., Kenangealem,E., Lindegardh,N., Penttinen,P., Laihad,F, Ebsworth,E.P., Anstey,N.M., and Tjitra, E. (2007). "Clinical and Pharmacological Determinants of the Therapeutic Response to Dihydroartemisinin-Piperaquine for Drug-Resistant Malaria". *Antimocrobial Agents and Chemotherapy*, Nov.2007, Vol.51, No.11, p.4090-4097.
Stable URL: http://aac.asm.org/content/51/11/4090.full.pdf
Accessed: 21/03/2012

Schoenfeld ,D (1982). "Partial residuals for the proportional hazards regression model". Biometrika, Vol. 69, No. 1. (Apr., 1982), pp. 239-241.
Stable URL: http://www.jstor.org/stable/2335876
Accessed: 14/12/2011 12:40

Terlouw, D.J., Nahlen, B.L.,Courval,J.M., Kariuki,S.K., Rosenberg,O.S., Oloo,A.J., Kolczak,M.S., Hawley,W.A., Lal,A.A., Kuile, F.O. (2003). "Sulfadoxine-Pyrimethamine in Treatment of Malaria in Western Kenya: Increasing Resistance and Underdosing". Antimicrobial agents and chemotherapy, sept. 2003, p. 2929–2932
Stable URL: http://aac.asm.org/content/47/9/2929.short
Accessed: 21/01/2012 12:40

6. Annex

A.1 List of available covariates

Description	Type
WWARN Status for Pf Adj	Primary Response
WWARN Status for Pf UnAdj	Primary Response
ETF	Secondary Response
LCF	Secondary Response
LPF	Secondary Response
LTF	Secondary Response
Early LTF (before D14, no PCR)	Secondary Response
History of Fever (0/1) at inclusion	Baseline Variable
Severe Malaria at inclusion	Baseline Variable
Haemoglobin at inclusion	Baseline Variable
Falciparum density at Inclusion	Baseline Variable
Gamf (/µL) at inclusion	Baseline Variable
Max Temp Day0	Baseline Variable
D0 Ht<20%	Baseline Variable
Age in Years	Available Variable
Gender	Available Variable
Weight	Available Variable
Antimalarial in last 28 days	Available Variable
Parasite density at Inclusion	Available Variable
Max Falciparum Asexual parasitaemia on Day1	Available Variable
Max Falciparum Asexual parasitaemia on Day2	Available Variable
Max Falciparum Asexual parasitaemia on Day3	Available Variable
Max Temp Day1	Available Variable
Max Temp Day2	Available Variable
Max Temp Day3	Available Variable
Dosing method (single day, broken down over days etc.)	Available Variable
Total mg/kg dose at each day of dosing regimen	Available Variable
Total mg/kg dose during course	Available Variable
