# Supplementary material for: The effect of dosing strategies on the therapeutic efficacy of artesunate-amodiaquine for uncomplicated malaria: a meta-analysis of individual patient data
Source: BMC Med. 2015 Mar 31;13:66. doi: 10.1186/s12916-015-0301-z (PMC4411752; doi:10.1186/s12916-015-0301-z)
Supplement: Additional file 6: — Text S6. Additional tables and figures. [file 12916_2015_301_MOESM6_ESM.docx]

**Table 1. Parasite Positivity Rates on day 1, 2 and 3 for different age categories.**

|  | FDC | | Co-blistered NFDC | | Loose NFDC-30 | | Loose NFDC-25 | |
| --- | --- | --- | --- | --- | --- | --- | --- | --- |
| Day1 |  |  |  |  |  |  |  |  |
| Age | **Positive** | **PPR [95% CI] ^a,b^** | **Positive** | **PPR [95% CI] ^a,b^** | **Positive** | **PPR [95% CI] ^a,b^** | **Positive** | **PPR [95% CI] ^a,b^** |
| <1 y | 96/142 | 67.6 [54.5-80.7] | 11/11 | 100[74.1-100]^c^ | 104/137 | 75.9 [64.8-87] | 22/27 | 81.5 [52.3-110.7] |
| 1 to <5 y | 1153/1564 | 73.7 [64.5-82.9] | 139/150 | 92.7 [75.8-109.6] | 482/745 | 64.7 [57.6-71.8] | 138/186 | 74.2 [63.6-84.7] |
| 5 to <12 y | 240/387 | 62 [47.8-76.2] | 53/129 | 41.1 [34.8-47.4] | 218/352 | 61.9 [46.5-77.4] | 37/66 | 56.1 [42-70.2] |
| ≥12 y | 208/425 | 48.9 [33.6-64.3] | 80/213 | 37.6 [27.9-47.2] | 56/158 | 35.4 [11.2-59.6] | 1/1 | 100 [5.1-100]^c^ |
| Overall | 1697/2518 | 67.4 [59.1-75.7] | 283/503 | 56.3 [29.1-83.4] | 656/1062 | 61.8 [54.7-68.9] | 198/280 | 70.7 [54.1-87.3] |
| Day2 |  |  |  |  |  |  |  |  |
| <1 y | 13/218 | 6 [1.4-10.5] | 5/83 | 6 [1.1-11] | 25/244 | 10.2 [5.1-15.4] | 13/172 | 7.6 [3.6-11.5] |
| 1 to <5 y | 176/2402 | 7.3 [3.2-11.4] | 24/597 | 4 [0.5-7.6] | 146/1473 | 9.9 [6.6-13.3] | 49/822 | 6 [1.8-10.2] |
| 5 to <12 y | 33/535 | 6.2 [2-10.4] | 10/213 | 4.7 [0.4-9] | 51/354 | 14.4 [6.1-22.7] | 12/234 | 5.1 [0.8-9.4] |
| ≥12 y | 29/569 | 5.1 [1.8-8.4] | 8/228 | 3.5 [0-7.3] | 9/161 | 5.6 [0-12.3] | 1/32 | 3.1 [0.7-5.5] |
| Overall | 246/3814 | 6.4 [3.5-9.4] | 47/1121 | 4.2 [1.6-6.8] | 192/1904 | 10.1 [7.5-13.2] | 75/1260 | 6 [2.8-9.1] |
| Day3 |  |  |  |  |  |  |  |  |
| <1 y | 3/235 | 1.3 [0-3.1] | 0/83 | 0[0-4.4]^c^ | 6/270 | 2.2 [0.7-3.8] | 2/176 | 1.1 [-0.8-3.1] |
| 1 to <5 y | 22/2472 | 0.9 [0.3-1.5] | 5/609 | 0.8 [0-1.7] | 26/1601 | 1.6 [0.8-2.5] | 5/835 | 0.6 [0-1.3] |
| 5 to <12 y | 3/631 | 0.5 [0-1.1] | 0/251 | 0[0-1.5]^c^ | 10/353 | 2.8 [0.8-4.9] | 2/241 | 0.8 [0-2.3] |
| ≥12 y | 1/568 | 0.2 [0-0.5] | 2/272 | 0.7 [0-1.6] | 3/162 | 1.9 [0-5.5] | 0/36 | 0[0-9.6]^c^ |
| Overall | 29/3906 | 0.7 [0.2-1.2] | 7/1215 | 0.6 [0.1-1] | 35/2058 | 1.7 [1.1-2.6] | 9/1288 | 0.7 [0.1-1.3] |

**^a^** Parasite positivity rates were calculated from those in whom a blood film was taken on that day; cases without a smear were removed from the denominator. Overall PPR on day1: 64.7% [95% CI: 58.5-71.0%], day2: 7.1% [95% CI: 5.2-9.0%] and day3: 1.0% [95% CI: 0.6-1.4%].

^b^ In studies with very frequent determination of parasitemia, measurements taken within ±3 hrs window of 24, 48 and 72 hours were classified as day1, 2 and 3 counts respectively. Patients with missing parasitaemia at a time point was considered positive (or negative) if the counts at both the time-points immediately before or after were both positive (or negative).

^c^Confidence Interval estimated using Wilson’s method as there were no cases with positive smear or all the data come from a single study. For the remaining estimates, confidence interval was estimated adjusting for within centre correlation.

**Table 2. Multivariable risk factors for parasite positivity on day1, day2 and day3.**

|  | Day1 | | | Day2 | | | Day3 | | |
| --- | --- | --- | --- | --- | --- | --- | --- | --- | --- |
| Multivariable Model | **N(n) ^a^** | **AOR [95% CI]** | ***p*-Value** | **N(n) ^a^** | **AOR [95% CI]** | ***p*-Value** | **N(n) ^a^** | **AOR [95% CI]** | ***p*-Value** |
| Parasitaemia (/10-fold rise) | 4685 (3032) | 3.87 [3.37-4.44] | <0.001 | 8348 (595) | 2.41 [2.02-2.89] | <0.001 | 8716 (90) | 1.76 [1.16-2.67] | 0.007 |
| mg/kg Artesunate dose (/unit) | 4685 (3032) | 0.98 [0.95-1.01] | 0.242 | 8348 (595) | 0.97 [0.93-1.01] | 0.151 | 8716 (90) | 0.92 [0.83-1.03] | 0.161 |
| Drug Formulation |  |  |  |  |  |  |  |  |  |
| FDC (reference) | 2518 (1697) | 1 | - | 3814 (246) | 1 | - | 3906 (29) | 1 | - |
| Coblisters | 503(283) | 1.37 [0.56-3.35] | 0.496 | 1121 (47) | 0.77 [0.38-1.57] | 0.475 | 1215 (7) | 0.95 [0.27-3.35] | 0.942 |
| Loose NFDC-25 | 280(198) | 2.83 [0.67-11.96] | 0.158 | 1256 (75) | 0.89 [0.35-2.27] | 0.809 | 1284 (9) | 1 [0.29-3.49] | 0.999 |
| Loose NFDC-30 | 1384(854) | 1.02 [0.45-2.29] | 0.968 | 2157(227) | 1.6 [0.84-3.02] | 0.149 | 2311(45) | 2.57 [1.12-5.94] | 0.027 |
| Age Category |  |  |  |  |  |  |  |  |  |
| ≥12 y | 797 (345) | 1 | - | 990 (47) | 1 | - | 1038(6) | 1 | - |
| <1 y | 313 (230) | 2.15 [1.42-3.26] | <0.001 | 703 (53) | 1.48 [0.87-2.51] | 0.149 | 750(11) | 1.49 [0.45-4.88] | 0.513 |
| 1 to <5 y | 2641(1909) | 2.1 [1.56-2.83] | <0.001 | 5230 (394) | 1.48 [0.95-2.29] | 0.081 | 5453(58) | 1.24 [0.44-3.48] | 0.687 |
| 5 to <12 y | 934 (548) | 1.39 [1.07-1.8] | 0.014 | 1425 (101) | 1.3 [0.85-2] | 0.232 | 1475 (15) | 1.17 [0.4-3.42] | 0.777 |

^a^ Number of patients for each variable/levels of factor with number of patients with positive smear shown inside the brackets.

**Table 3. Sensitivity Analysis by removing 1 study site at a time**

| Variable | Jacknifed Estimator  [95% CI] ^a^ | Multivariable Estimates (Table 5) | Coefficient of Variation (%) ^b^ |
| --- | --- | --- | --- |
| Baseline Parasitaemia (per 10-fold) | 1.38[1.10-1.74] | 1.39 [1.1-1.74] | 2.10% |
| mg/kg AQ Dose (every 5 unit) | 0.94[0.84-1.06] | 0.94 [0.84-1.05] | 1.21% |
| Age Category |  |  |  |
| ≥12 y (reference) | 1 |  |  |
| <1 | 3.90[1.73-8.85] | 3.93 [1.76-8.79] | 6.60% |
| 1 to <5 | 4.33[2.08-9.04] | 4.47 [2.18-9.19] | 5.58% |
| 5 to <12 | 1.99[0.93-4.28] | 2.03 [0.96-4.28] | 6.01% |
| Drug Formulation |  |  |  |
| FDC (reference) |  |  |  |
| Co-blistered NFDC | 1.39[0.75-2.58] | 1.38 [0.75-2.57] | 3.17% |
| Loose: Target AQ dose 25 mg/kg | 3.51[2.03-6.08] | 3.51 [2.02-6.12] | 3.93% |
| Loose: Target AQ dose 30 mg/kg |  |  |  |
| In Rukara/Kailahun/Kisumu | 7.76[4.16-14.50] | 7.75 [4.07-14.76] | 3.48% |
| Rest of the sites | 1.5[0.92-2.46] | 1.47 [0.91-2.38] | 3.67% |
| Region |  |  |  |
| Africa(reference) |  |  |  |
| Asia | 7.30[3.41-15.62] | 7.39 [3.45-15.86] | 5.25% |

^a^ Jacknifing was carried out by removing one study site at a time. The estimates presented are mean and associated 95% confidence intervals.

^b^The coefficient of variation calculated as standard deviaton divided by the mean estimates from the jacknifed coefficients.

**Table 4: Assessment of proportional hazard within each study**

| Study ^c^ | Failures by day 28 | Baseline Parasitaemia | Mg/kg AQ dose | <1 y | 1 to <5 y | 5 to <12 y | Global Test |
| --- | --- | --- | --- | --- | --- | --- | --- |
| Barennes-2004 | 0 | - | - | - | - | - | - |
| Faye-2010 | 0 | - | - | - | - | - | - |
| Grandesso-2006 | 0 | - | - | - | - | - | - |
| Guthmann-2006 | 0 | - | - | - | - | - | - |
| Jullien-2010 | 0 | - | - | - | - | - | - |
| Juma-2005 ^a^ | 0 | - | - | - | - | - | - |
| Ndiaye-2011 | 0 | - | - | - | - | - | - |
| Osorio-2007 | 0 | - | - | - | - | - | - |
| Sinou-2009 | 0 | - | - | - | - | - | - |
| Temu-2010 ^a^ | 0 | - | - | - | - | - | - |
| Adjuik-2002 | 1 | - | - | - | - | - | - |
| Bonnet-2007 | 1 | - | - | - | - | - | - |
| Espié-2012 | 1 | - | - | - | - | - | - |
| Guthmann-2005 | 1 | - | - | - | - | - | - |
| Schramm-2013 | 1 | - | - | - | - | - | - |
| van den Broek-2005 | 1 | - | - | - | - | - | - |
| Brasseur-2011 | 2 | - | - | - | - | - | - |
| Gaye-2010b ^a^ | 2 | - | - | - | - | - | - |
| Laminou-2011 ^a^ | 2 | - | - | - | - | - | - |
| Sanofi-2013 ^a^ | 2 | - | - | - | - | - | - |
| Dorsey-2007 | 3 | - | - | - | - | - | - |
| Hamour-2005 | 3 | - | - | - | - | - | - |
| Karema-2006 | 3 | - | - | - | - | - | - |
| Ndiaye-2009 | 3 | - | - | - | - | - | - |
| Grandesso-2003 ^a^ | 4 | - | - | - | - | - | - |
| Menan-2012 ^a^ | 4 | - | - | - | - | - | - |
| Sagara-2012 | 4 | - | - | - | - | - | - |
| Staedke-2004 | 4 | - | - | - | - | - | - |
| Anvikar-2012 | 5 | 0.477 | 0.562 | - | - | - | 0.646 |
| Hasugian-2007 | 5 | 0.728 | 0.023 | - | - | - | 0.070 |
| Ménard-2007 | 5 | 0.376 | 0.030 | - | - | - | 0.073 |
| Rwagacondo-2004 | 5 | 0.683 | 0.163 | - | - | - | 0.314 |
| Swarthout-2006 | 5 | 0.192 | 0.922 | - | - | - | 0.417 |
| Bukirwa-2006 ^b^ | 6 | D/C | D/C | D/C | D/C | D/C | D/C |
| Faucher-2009 | 6 | 0.625 | 0.160 | - | - | - | 0.365 |
| Kayentao-2009 | 6 | 0.150 | 0.948 | - | - | - | 0.351 |
| Nikiema-2010 ^a^ | 8 | 0.173 | 0.976 | - | - | - | 0.393 |
| Smithuis-2010 | 10 | 0.518 | 0.432 | - | 0.866 | 0.467 | 0.564 |
| Thwing-2009 | 10 | 0.219 | 0.092 | - | 0.075 | - | 0.098 |
| Mårtensson-2005 | 11 | 0.982 | 0.841 | - | 0.718 | 0.084 | 0.117 |
| Sirima-2009 | 17 | 0.474 | 0.580 | - | - | - | 0.642 |
| The 4ABC StudyGroup-2011 | 18 | 0.604 | 0.756 | - | - | - | 0.825 |
| Yeka-2005 | 47 | 0.611 | 0.617 | - | - | - | 0.807 |

^a^ Unpublished studies

^b^ D/C= Model didn’t converge

^c^ The assumption of proportion hazard was tested for each of the individual study. This couldn’t be assessed in studies with <5 failures. Because of the low number of failures within each study, comparison between different age categories lacked power.

**Table 5. Risk factors for Adverse Events**

1. **Risk factor for Neutropeania within 28 days of follow-up in patients with normal neutrophils at enrolment**

| Variable ^a^ | N(n)^b^ | Adjusted OR [95% CI] | *p*-Value |
| --- | --- | --- | --- |
| mg/kg Amodiaquine dose (/5 unit) | 489(103) | 1.16 [0.93-1.43] | 0.181 |
| Age Category |  |  |  |
| ≥12 y (reference) | 79 (30) | 1 |  |
| <1 y | 50 (15) | 0.33 [0.03-3.45] | 0.354 |
| 1 to <5 y | 255 (44) | 0.14 [0.01-1.36] | 0.090 |
| 5 to <12 y | 105 (14) | 0.37 [0.04-3.4] | 0.378 |
| Drug Formulation ^c^ |  |  |  |
| FDC (reference) | 23 (12) | 1 |  |
| Co-blistered NFDC | 26 (11) | 0.59 [0.19-1.85] | 0.362 |
| Loose NFDC-25 | 127 (6) | 0.19 [0-10.56] | 0.417 |
| Loose NFDC-30 | 313 (74) | 0.37 [0.02-8.85] | 0.542 |

^a^Neutropenia defined as ≤1200 neutrophils/μL for <12 years and ≤1500 neutrophils/μL for ≥12 years.

^b^ Number of patients [n] for each variable/levels of factor with number of patients developing neutropeania within day 28 in those who had normal values at baseline.

^c^ None of the pairwise comparisions between the different formulations were statistically significant (all *p*>0.05).

1. **Risk factors for Anaemia within 28 days of follow-up in patients with normal haemoglobin at enrolment**

| Variable ^a^ | N(n) ^b^ | Adjusted OR [95% CI] | *p*-Value |
| --- | --- | --- | --- |
| mg/kg Amodiaquine dose (5 unit) | 2557 (590) | 1.05 [0.94-1.18] | 0.3471 |
| Baseline Parasitaemia (10-fold) |  | 1.64 [1.33-2.03] | *p*<0.001 |
| Age Category |  |  |  |
| ≥12 y (reference) | 468 (39) |  |  |
| <1 y | 129 (64) | 42.82 [20.11-91.18] | *p* <0.001 |
| 1 to <5 y | 1558 (437) | 9.6 [5.12-18] | *p* <0.001 |
| 5 to <12 y | 402 (50) | 3.53 [2.08-6.01] | *p* <0.001 |
| Drug Formulation |  |  |  |
| FDC (reference)^c^ | 1077 (322) |  |  |
| Co-blistered NFDC | 232 (39) | 0.66 [0.2-2.13] | 0.483 |
| Loose NFDC-25 | 525(69) | 0.22 [0.05-0.85] | 0.027 |
| Loose NFDC-30 | 723 (160) | 0.33 [0.12-0.91] | 0.032 |
| New Infection |  |  |  |
| New Infection during the follow-up | 2557 (443) | 1.47 [1.07-2.01] | 0.018 |

^a^Anaemia defined as haemoglobin < 10 g/dL.

^b^ Number of patients [n] for each variable/levels of factor with number of patients developing anaemia within day 28 in those who had normal values at baseline.

^c^Pairwise comparisions:

+Compared to Co-blistered NFDC, none of the formulations were significantly different (all *p*>0.05)

+Compared to Loose NFDC-25, AOR=4.64[95% CI: 1.18-18.19], *p*=0.027 for FDC. All other comparison were not significant (*p*>0.05)

+Compared to Loose NFDC-30, AOR=3.03[95% CI: 1.09-8.42], *p*=0.032 for FDC; All other comparison were not significant (*p*>0.05)

1. **Risk factors for Diarrhoea between Day1-Day7**

| Variable | N(n) ^a^ | Adjusted OR [95% CI] | *p*-Value |
| --- | --- | --- | --- |
| mg/kg Amodiaquine dose (5 unit) | 3821(290) | 1.16 [1.08-1.25] | *p*<0.001 |
| Age Category |  |  |  |
| ≥12 y (reference) | 469 (33) |  |  |
| <1 y | 282 (52) | 3.81 [1.77-8.18] | *p*<0.001 |
| 1 to <5 y | 2565 (189) | 1.15 [0.58-2.29] | 0.682 |
| 5 to <12 y | 505 (16) | 0.63 [0.31-1.3] | 0.210 |
| Drug Formulation ^b^ |  |  |  |
| FDC (reference) | 2517 (151) |  |  |
| Co-blistered NFDC | 487 (55) | 0.82 [0.53-1.27] | 0.369 |
| Loose NFDC-25 ^c^ | - | - | - |
| Loose NFDC-30 | 817 (84) | 1.95 [0.85-4.48] | 0.116 |

^a^ Number of patients [n] for each variable/levels of factor with number of patients reporting diarrhoea between day1-day7.

^b^ None of the pairwise comparison between different formulations were significant (all *p*>0.05).

^c^ Data not available for this formulation.

1. **Risk factors for Vomiting between Day1-Day7**

| Variable | N(n)^a^ | Adjusted OR [95% CI] | *p*-Value |
| --- | --- | --- | --- |
| mg/kg Amodiaquine dose (5 unit) | 3721 (410) | 1.2 [1.11-1.3] | *p*<0.001 |
| Age Category |  |  |  |
| ≥12 y (reference) | 387 (94) |  |  |
| <1 y | 287 (19) | 0.49 [0.25-0.95] | 0.034 |
| 1 to <5 y | 2611 (228) | 0.46 [0.3-0.7] | *p*<0.001 |
| 5 to <12 y | 436 (69) | 0.53 [0.35-0.81] | 0.003 |
| Drug Formulation ^b^ |  |  |  |
| FDC (reference) | 2330 (256) |  |  |
| Co-blistered NFDC | 472 (29) | 1.10 [0.53-2.28] | 0.793 |
| Loose NFDC-25 | 101 (9) | 1.56 [0.26-9.26] | 0.626 |
| Loose NFDC-30 | 818 (116) | 1.27 [0.62-2.59] | 0.511 |

^a^ Number of patients [n] for each variable/levels of factor with number of patients who vomited at least once between day1-day7.

^b^ None of the pairwise comparison between different formulations were significant (all *p*>0.05).

1. **Risk factors for Acute Drug Vomiting**

| Variable | N(n)^a^ | Adjusted OR [95% CI] | *p*-Value |
| --- | --- | --- | --- |
| mg/kg Amodiaquine dose (5 unit) | 2351 (294) | 1.23 [1.12-1.36] | *p*<0.001 |
| Age Category |  |  |  |
| ≥12 y (reference) | 278 (11) |  |  |
| <1 y | 126 (27) | 6.35 [2.87-14.05] | *p*<0.001 |
| 1 to <5 y | 1655 (230) | 3.47 [1.79-6.73] | *p*<0.001 |
| 5 to <12 y | 292 (26) | 2.02 [0.96-4.25] | 0.066 |
| Drug Formulation |  |  |  |
| FDC (reference) | 2007 (259) |  |  |
| Co-blistered NFDC ^b^ | - |  |  |
| Loose NFDC-25 ^b^ | - |  |  |
| Loose NFDC-30 | 344(35) | 0.68 [0.41-1.11] | 0.124 |

^a^ Number of patients [n] for each variable/levels of factor with number of patients who vomiting at least one dose of the medication within an hour of the dose administration during the course of treatment

^b^ Data on dose vomiting not available for this formulation.

**Table 6: Bias Assessment of the studies where Individual patient were not available for analysis**

| Reference | Year | Country | Age -Range  (months) | Formulation | N | Supervision | Target Dose  of AQ | Target Dose  of AS | PP PCR Corrected  ACPR at day28 | Simulated ACPR  [95% CI] ^a^ |
| --- | --- | --- | --- | --- | --- | --- | --- | --- | --- | --- |
| Ali-2013 | 2013 | Cameroon | 6–168 | Co-blistered NFDC | 73 | Full | 30 mg/kg | 12 mg/kg | 0.964 | 0.975[0.939-1] |
| Asih-2009 | 2009 | Indonesia | 6–84 | Loose-30 | 103 | Full | 30 mg/kg | 12 mg/kg | 1 | 0.964[0.928-1] |
| Ayede-2010 | 2010 | Nigeria | 12–156 | FDC | 238 | Full | 30 mg/kg | 12 mg/kg | 0.979 | 0.979[0.961-0.997] |
| Charle-2013 | 2013 | Equatorial Guinea | 6–59 | Loose-30 | 122 | Full | 30 mg/kg | 12 mg/kg | 0.973 | 0.966[0.934-0.998] |
| De la Hoz Restrepo-2012 | 2012 | Colombia | >216 | FDC | 105 | Full | 30 mg/kg | 12 mg/kg | 1 | 0.994[0.979-1] |
| Dorkenoo-2012 | 2012 | Togo | 6–59 | Loose-30 (2005-07) | 485 | Full | 30 mg/kg | 12 mg/kg | 0.989 | 0.967[0.951-0.983] |
| Dorkenoo-2012 | 2012 | Togo | 6–59 | Co-Blisters (2009) | 166 | Full | 30 mg/kg | 12 mg/kg | 0.994 | 0.971[0.945-0.997] |
| Falade-2008b | 2008 | Ghana | 6–120 | Loose-30 | 66 | Full | 30 mg/kg | 12 mg/kg | 1 | 0.971[0.929-1] |
| Falade-2014 | 2014 | Nigeria | 6–84 | Loose-30 | 53 | Full | 30 mg/kg | 12 mg/kg | 0.958 (46/48) | 0.942[0.879-1] |
| Faye-2012 | 2012 | Multicentred | 7–84 | FDC | 246 | Full | 30 mg/kg | 12 mg/kg | 0.987 | 0.979[0.962-0.997] |
| Fehintola-2008 | 2008 | Nigeria | Children | - | 61 | - | - | - | - | - |
| Gbotosho-2011a | 2011 | Nigeria | ≤ 144 | FDC | 99 | Full | - | - | 1 | 0.979[0.951-1] |
| Gbotosho-2011a | 2011 | Nigeria | ≤ 144 | Co-blistered NFDC | 104 | Full | - | - | 0.99 | 0.974[0.944-1] |
| Gbotosho-2012a | 2012 | Nigeria | ≤ 180 | FDC | - | - | - | - | - | - |
| Ibrahium-2007 | 2007 | Sudan | - | - | 42 | - | 30 mg/kg | 12 mg/kg | 0.952 | 0.971[0.922-1] |
| Kabanywanyi-2007 | 2007 | Tanzania | 6–59 | Loose-25 | 76 | Full | 25 mg/kg | 12 mg/kg | 0.938 | 0.921[0.860-0.983] |
| Kobbe-2008 ^b^ | 2008 | Ghana | 6–59 | Co-Blistered NFDC | 117 | Unsupervised | - | - | 0.927 (89/96) | 0.971[0.942-1] |
| Koram-2005 | 2005 | Ghana | 6–59 | Loose-25 | 54 | Full | 25 mg/kg | 12 mg/kg | 1 | 0.920[0.848-0.992] |
| Michael-2010 | 2010 | Nigeria | 12–132 | Loose-30 | 96 | Full | 30 mg/kg | 12 mg/kg | 0.978 | 0.967[0.932-1] |
| Nambei-2013 | 2013 | CAR | 6–59 | - | 60 | - | - | - | 1 | - |
| Ndounga-2013 ^b^ | 2013 | Republic of Congo | 8–648 | Co-blistered NFDC | 197 | Full | 30 mg/kg | 12 mg/kg | 0.944 | 0.978[0.958-0.998] |
| Oduro-2008 | 2008 | Ghana | 6–120 | - | 154 | Full | 30 mg/kg | 12 mg/kg | - | - |
| Oduro-2008 | 2008 | Ghana | 6–120 | - | 154 | Unsupervised | 30 mg/kg | 12 mg/kg | - | - |
| Ojurongbe O-2013 | 2013 | Nigeria | 6–144 | FDC | 71 | Full | 30 mg/kg | 12 mg/kg | 1 | 0.978[0.945-1] |
| Onyamboko-2014 | 2014 | DRC | 3–59 | FDC | 228 | Full | ~30.6 mg/kg | ~11.4 mg/kg | 0.981 | 0.979[0.960-0.997] |
| Owusu-Agyei-2008 ^b^ | 2008 | Ghana | Children | Co-blistered NFDC | 151 | Full | - | - | 0.934 | 0.971[0.945-0.998] |
| Sahr-2013 | 2013 | Sierra Leone | <60 | - | 109 | Full | - | - | 1 | - |
| Sowunmi-2005 | 2005 | Nigeria | ≤ 144 | Loose-30 | 104 | Full | 30 mg/kg | 12 mg/kg | 1 | 0.970[0.939-1] |
| Sowunmi-2012 | 2012 | Nigeria | - | - | 68 | - | - | - | - | - |
| Sowunmi-2013 | 2013 | Nigeria | - | - | 127 | - | - | - | 1 | - |
| Sylla K-2013 | 2013 | Senegal | All Age | FDC | 180 | Full | 30 mg/kg | 12 mg/kg | 0.994 | 0.981[0.962-1] |
| Tahar-2014 | 2014 | Cameroon | 6–60 | Loose-30 | 70 | Full | 30 mg/kg | 12 mg/kg | 0.968 | 0.940[0.884-0.996] |
| Thanh-2009 | 2009 | Vietnam | 144–720 | Loose-30 | 56 | Full | 31.8 mg/kg | 13.2 mg/kg | 0.98 (48/49) | 0.991[0.965-1] |
| Thanh-2012 | 2012 | Vietnam | 60–720 | FDC | 65 | Full | ~36 mg/kg | ~13.2 mg/kg | 0.98 (Day42) | 0.983[0.951-1] |
| Tinto-2014^b^ | 2014 | Burkina Faso | <60 | Co-blistered NFDC | 166 | Unsupervised | 22.5 - 45 mg/kg | 8.4 to 16.5 mg/kg | 0.897 | 0.971[0.945-0.996] |
| Warsame-2009 (Jamame) | 2009 | Somalia | 6–120 | Loose-30 | 95 | Full | 30 mg/kg | 12 mg/kg | 0.978 | 0.970[0.936-1] |
| Warsame-2009 (Janale) | 2009 | Somalia | 6–120 | Loose-30 | 103 | Full | 30 mg/kg | 12 mg/kg | 0.981 | 0.969[0.938-1] |
| Whegang-2010 | 2010 | Cameroon | 6–660 | Loose-30 | 199 | Full | 30 mg/kg | 12 mg/kg | 0.95 (189/199) | 0.971[0.949-0.995] |

^a^The PCR-Corrected efficacy estimates for the given age-range from the study not available was estimated from the pooled analysis database ($\theta_{i}$). A total of $n$ (n= study sample size) patients were simulated from a binomial distribution (assuming a simple case of no censoring structure) with probability of success, $\theta_{i}$. A study with a sample size *n* was simulated 1000 times from which mean cure rate and associated 95% Confidence Interval was estimated.

^b^ For theset studies, the reported cure rate for the study in publication don’t fall within the associated 95% confidence interval derived from simulated data, suggesting that these studies are different from the rest of the studies included in the pooled analysis. Although the reported cure rate doesn’t fall within the simulated confidence interval, the estimates are nearly overlapping with the lower bound of 95% CI suggesting the bias is small. Similarly, Tinto-2014 and Kobbe-2008 were effective trials with dosing regimen not supervised which could probably explain lower efficacy reported in those studies.

**Table 7: Assessment of the between study heterogeneity**

| **Measure of between-study Heterogeneity ^a^** | **Dose** | **Baseline Parasitaemia** |
| --- | --- | --- |
| I^2^ statistics [95% CI] | 3.9% [0%; 32.7.8%] | 25.8% [0%; 52.8%] |
| Cochran’s Q | Q= 30.18 (*p*=0.41) | Q= 39.09 (*p*=0.10) |

^a^In addition of specifying the estimated variation of random effects in footnotes of the multivariable cox model (in Table 5), we have performed a meta-analysis of log-transformed hazards ratio for two continuous predictors: parasitaemia and dose, and give the estimate of between study heterogeneity summarised by I^2^ and Chochran’s Q statistics. However, such assessment of between study heterogeneity wasn’t possible for the categorical covariates because of non-availability of all the levels of categories within each study.

**Table 8: Loss to Follow-Up (LFU) within each study**

| Title | Total Patient | Completed the day28 follow-up | LFU by day28 | LFU (%) |
| --- | --- | --- | --- | --- |
| Adjuik-2002 | 390 | 338 | 52 | 13.3% |
| Anvikar-2012 | 199 | 194 | 5 | 2.5% |
| Barennes-2004 | 32 | 27 | 5 | 15.6% |
| Bonnet-2007 | 110 | 107 | 3 | 2.7% |
| Brasseur-2011 | 276 | 145 | 131 | 47.5% |
| Bukirwa-2006 | 203 | 201 | 2 | 1.0% |
| Dorsey-2007 | 145 | 133 | 12 | 8.3% |
| Espié-2012 | 149 | 135 | 14 | 9.4% |
| Faucher-2009 | 94 | 87 | 7 | 7.4% |
| Faye-2010 | 155 | 148 | 7 | 4.5% |
| Gaye-2010b | 129 | 119 | 10 | 7.8% |
| Grandesso-2003 | 86 | 84 | 2 | 2.3% |
| Grandesso-2006 | 123 | 109 | 14 | 11.4% |
| Guthmann-2005 | 96 | 81 | 15 | 15.6% |
| Guthmann-2006 | 68 | 63 | 5 | 7.4% |
| Hamour-2005 | 71 | 71 | 0 | 0.0% |
| Hasugian-2007 | 93 | 76 | 17 | 18.3% |
| Jullien-2010 | 51 | 47 | 4 | 7.8% |
| Juma-2005 | 201 | 201 | 0 | 0.0% |
| Karema-2006 | 251 | 249 | 2 | 0.8% |
| Kayentao-2009 | 128 | 126 | 2 | 1.6% |
| Laminou-2011 | 80 | 69 | 11 | 13.8% |
| Mårtensson-2005 | 202 | 200 | 2 | 1.0% |
| Menan-2012 | 110 | 97 | 13 | 11.8% |
| Ménard-2007 | 332 | 314 | 18 | 5.4% |
| Ndiaye-2009 | 625 | 591 | 34 | 5.4% |
| Ndiaye-2011 | 179 | 178 | 1 | 0.6% |
| Nikiema-2010 | 527 | 510 | 17 | 3.2% |
| Osorio-2007 | 37 | 30 | 7 | 18.9% |
| Rwagacondo-2004 | 157 | 153 | 4 | 2.5% |
| Sagara-2012 | 230 | 219 | 11 | 4.8% |
| Sanofi-2013 | 203 | 192 | 11 | 5.4% |
| Schramm-2013 | 147 | 144 | 3 | 2.0% |
| Sinou-2009 | 13 | 11 | 2 | 15.4% |
| Sirima-2009 | 878 | 755 | 123 | 14.0% |
| Smithuis-2010 | 142 | 136 | 6 | 4.2% |
| Staedke-2004 | 130 | 126 | 4 | 3.1% |
| Swarthout-2006 | 82 | 75 | 7 | 8.5% |
| Temu-2010 | 99 | 95 | 4 | 4.0% |
| The 4ABC StudyGroup-2011 | 981 | 890 | 91 | 9.3% |
| Thwing-2009 | 101 | 99 | 2 | 2.0% |
| van den Broek-2005 | 87 | 83 | 4 | 4.6% |
| Yeka-2005 | 714 | 688 | 26 | 3.6% |
| Overall | **9106** | **8396** | **710** | **7.8%** |

**Table 9: Multiple Imputation Methods**

| ***Variable ^a^*** | ***Analysis*** *^b^* | ***Fraction of Missing Information (FMI)*** | ***Hazards Ratio [95% CI]*** |
| --- | --- | --- | --- |
| ***Baseline Haemoglobin*** | *i. Complete Case Analysis* | *-* | *0.94 [0.88-1.02]* |
| *(33% missing)* | *ii. Multiple Imputation* |  |  |
|  | *m =100* | *0.21* | *0.96[0.88-1.03]* |
| ***Baseline Anaemia*** | *i. Complete Case Analysis* | *-* | *1.35 [1.02-1.78]* |
| *(33% missing)* | *ii. Multiple Imputation* |  |  |
|  | *m =100* | *0.10* | *1.29[0.98- 1.72]* |
| ***Baseline Gametocytaemia*** | *i. Complete Case Analysis* | *-* | *1.01[0.52- 1.97]* |
| *(48% missing)* | *ii. Multiple Imputation* |  |  |
|  | *m =100* | *0.44* | *0.97[0.58-1.62]* |

^a^Age, parasitaemia and gender were used as predictors for haemoglobin and baseline anaemia. For baseline gametocytaemia, predictors used were age, parasitaemia, baseline haemoglobin and region.

*^b^*Multiple Imputation using chained equations were carried out using *MICE* package in R to handle the missing information on haemoglobin and baseline gametocytaemia. The adequate number of imputations (*m*) was computed based on fraction of missing information (FMI) accepting 5% loss in efficiency using $m\geq100*FMI$. The estimates from the 100 imputation runs were summarised using method proposed by Rubin (1987).

**Figure 1: Imputing missing baseline haemoglobin and baseline gametocytaemia and their affect in multivariable risk factor model**

**
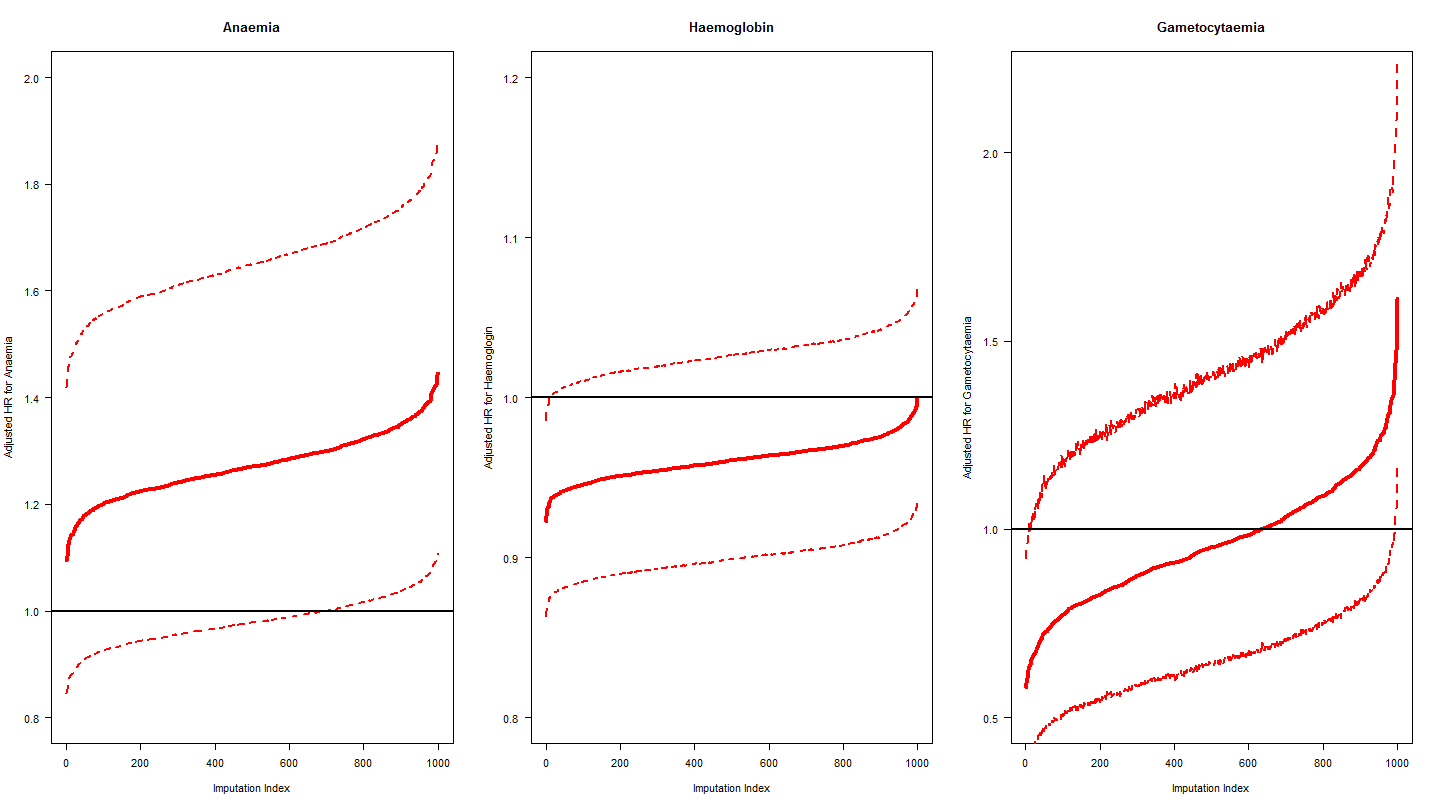
**

**Legend**: Random imputation was performed to assess the effect of anaemia, haemoglobin and baseline gametocytemia. This was repeated 1000 times. The bold line is the adjusted hazard ratio obtained from multivariable model adjusted for age-category, mg/kg AQ dose, baseline parasitaemia and drug formulations. The outer dotted lines are the lower and upper bounds of associated 95% confidence interval. Both parameters were found not to be significant in multivariable model.

**Figure2: Bootstrap Distribution of Parameter Estimates.**

**
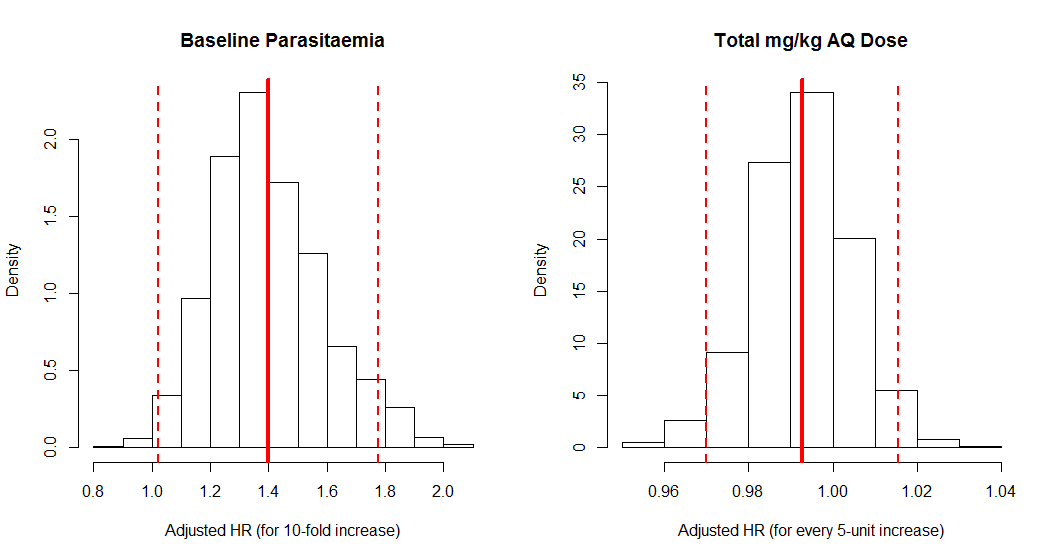
**

**
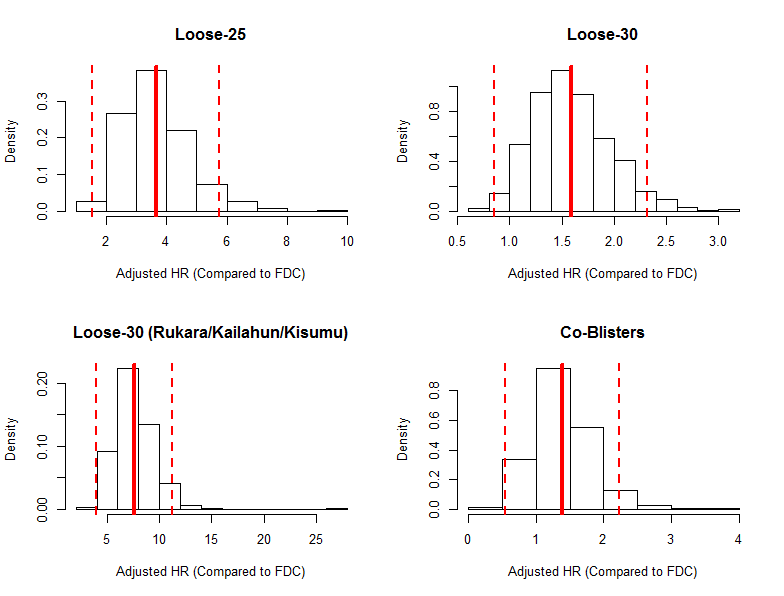
**


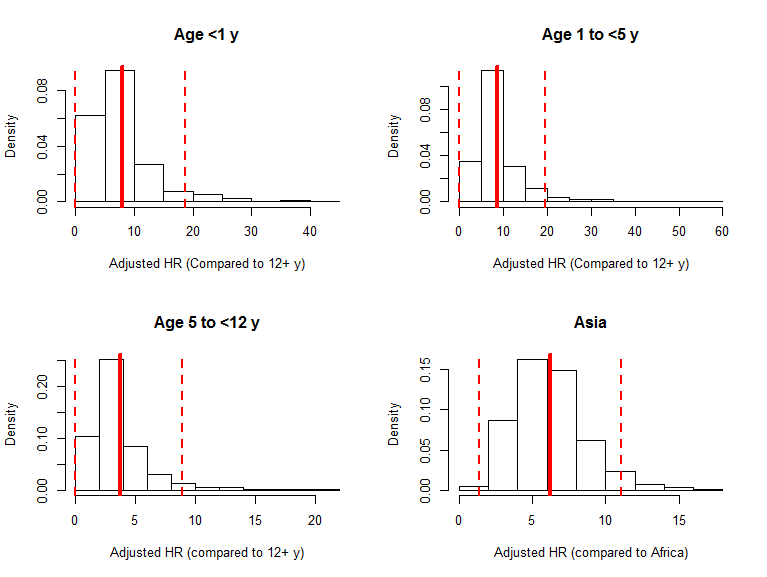


**Legend**: Bootstrapping was performed by taking 1000 random samples from the dataset. The bold line represents the mean estimates and the dashed lines are associated 95% confidence interval obtained from the bootstrap samples.
